# Unique structural features of a bacterial autotransporter adhesin suggest mechanisms for interaction with host macromolecules

**DOI:** 10.1038/s41467-019-09814-6

**Published:** 2019-04-29

**Authors:** Jason J. Paxman, Alvin W. Lo, Matthew J. Sullivan, Santosh Panjikar, Michael Kuiper, Andrew E. Whitten, Geqing Wang, Chi-Hao Luan, Danilo G. Moriel, Lendl Tan, Kate M. Peters, Minh-Duy Phan, Christine L. Gee, Glen C. Ulett, Mark A. Schembri, Begoña Heras

**Affiliations:** 1 Department of Biochemistry and Genetics, La Trobe Institute for Molecular Science, La Trobe University, Melbourne, 3086 VIC Australia; 2 School of Chemistry and Molecular Biosciences, and Australian Infectious Diseases Research Centre, The University of Queensland, Brisbane, 4072 QLD Australia; 3 School of Medical Science, and Menzies Health Institute Queensland, Griffith University, Gold Coast, 4222 QLD Australia; 4 Macromolecular Crystallography, Australian Synchrotron, Clayton, 3168 VIC Australia; 5 Department of Molecular Biology and Biochemistry, Monash University, Melbourne, 3800 VIC Australia; 6 Molecular & Materials Modelling group Data61, CSIRO, Docklands, Melbourne, 8012 VIC Australia; 7 Australian Centre for Neutron Scattering, Australian Nuclear Science and Technology Organisation, Lucas Heights, 2234 NSW Australia; 8 High Throughput Analysis Laboratory and Department of Molecular Biosciences, Northwestern University, Chicago, 60208 IL USA

**Keywords:** Structural biology, Bacterial pathogenesis, Bacterial structural biology, Pathogens

## Abstract

Autotransporters are the largest family of outer membrane and secreted proteins in Gram-negative bacteria. Most autotransporters are localised to the bacterial surface where they promote colonisation of host epithelial surfaces. Here we present the crystal structure of UpaB, an autotransporter that is known to contribute to uropathogenic *E. coli* (UPEC) colonisation of the urinary tract. We provide evidence that UpaB can interact with glycosaminoglycans and host fibronectin. Unique modifications to its core β-helical structure create a groove on one side of the protein for interaction with glycosaminoglycans, while the opposite face can bind fibronectin. Our findings reveal far greater diversity in the autotransporter β-helix than previously thought, and suggest that this domain can interact with host macromolecules. The relevance of these interactions during infection remains unclear.

## Abstract (web summary)

Autotransporter proteins are localised to the bacterial surface and promote colonisation of host epithelial surfaces. Here, the authors present the crystal structure of autotransporter UpaB and show evidence for distinct binding sites for glycosaminoglycans and host fibronectin.

## Introduction

A key factor in the establishment of infections by most bacterial pathogens is their adherence to host epithelial cells^1,2^. Autotransporters (ATs) are the largest group of outer membrane and secreted proteins in bacteria and play important roles in virulence, including promoting adhesion^3^. ATs share a common domain organisation consisting of a Sec-dependent signal sequence, a passenger or α-domain and a C-terminal translocator β-domain^4^. The signal sequence and β-domain are required for transport of the α-domain through the inner and outer membranes, respectively. The α-domain is the functional portion of the protein and can drive phenotypes including cytotoxicity, aggregation, adhesion and/or invasion, features that enhance bacterial virulence, colonisation, biofilm formation, persistence and resistance to host innate defence mechanisms^3–5^. In an era of increasing antimicrobial resistance, a greater understanding of the mechanisms by which ATs augment bacterial pathogenesis is required if we are to develop new strategies to combat infections caused by multidrug-resistant pathogens.

Despite the abundance of AT genes in the GenBank sequence database and the importance of these proteins in bacterial pathogenesis, structural information about AT α-domains remains limited. To date, only 11 α-domains and some small fragments of trimeric ATs (a separate subfamily of ATs that are obligate trimers^6–8^) have been structurally characterised, and consequently, AT molecular mechanisms of action are largely unknown.

Based on limited structural information, AT α-domains adopt a general architecture comprising a long narrow right-handed β-helix that is embellished with loops and/or other small domains (e.g. trypsin-like domains) that confer different functional properties^9–12^. One of the best characterised ATs is the antigen 43 (Ag43) protein, which belongs to the largest and most diverse AT subfamily, the AIDA-I type class^13^. Ag43 is the only member with a known mechanism of action, whereby a head to tail dimerisation of Ag43 monomers on the surface of adjacent bacterial cells promotes aggregation and biofilm formation via a molecular ‘velcro-like’ mechanism^14–17^.

The surface-exposed α-domains of different AIDA-I type ATs exhibit extensive sequence variation^13^. An example of this is represented by UpaB from uropathogenic *Escherichia coli* (UPEC), the major aetiologic agent of urinary tract infection and a primary cause of sepsis^18^. UpaB is an AT that mediates UPEC adherence to extracellular matrix (ECM) proteins and enhances UPEC colonisation of the urinary tract^19^. Here we determine the structure of UpaB at high resolution and reveal that it adopts a unique architecture potentially comprising two distinct binding sites. One binding site is formed by significant extensions to its β-helix that form a groove that can interact with glycosaminoglycans. On its opposite face, a second binding region can interact with human fibronectin (FN) type III (FnIII). Our results suggest that the AT β-helix may have diverse roles in addition to acting as a structural scaffold^5^.

## Results

### Characterisation of UpaB reveals that it does not exhibit self-association properties

**Fig. 1:**
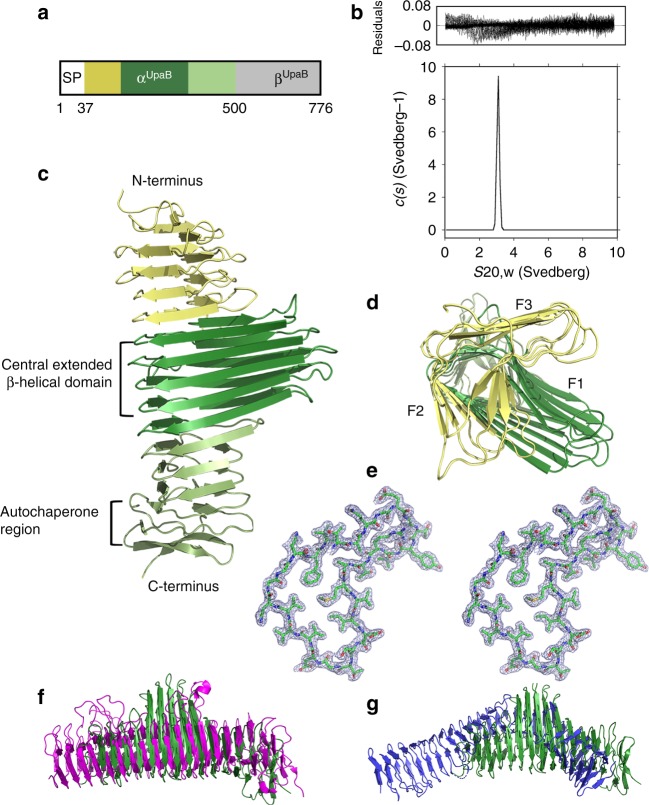
The structure of the UpaB functional α-domain (α^UpaB^). **a** Domain organisation of UpaB comprising an N-terminal signal sequence (SP; residues 1–37), an α-domain (α^UpaB^; residues 38–500) and a β-domain (β^UpaB^; residues 501–776). **b** Analytical ultracentrifugation sedimentation velocity analysis of α^UpaB^. The continuous standardised sedimentation distribution [*c*(*s*)] shows that UpaB at 2.2 mg ml^−1^ exists as a 3.1 *s*_20,w_ monomer. **c** Cartoon representation of the α^UpaB^ structure, including **d** top view. The central domain consisting of extended β-strands is shown in dark green. The N-terminal and C-terminal β-helical domains are shown in yellow and light green, respectively. The top view has F1, F2 and F3 faces shown. **e** Stereo view of the 2*F*_o_−*F*_c_ electron density map contoured at 1σ of the cross-section of the α^UpaB^ β-helix. Structural comparison of UpaB (green) with the α-domain of **f** pertactin (from *B. pertussis*; magenta; PDB 1DAB) and **g** Ag43a (from UPEC; blue; PDB 4KH3)

AIDA-I-type ATs including Ag43, TibA and AIDA-I belong to a group of self-associating ATs that promote bacterial aggregation and biofilm formation^20^. In the case of Ag43, aggregation is mediated via a series of hydrogen bonds, hydrophobic interactions and Van der Waals forces that drive a head-to-tail dimersation of Ag43 monomers on the surface of adjacent cells^17^. We previously showed that UpaB from the UPEC reference strain CFT073 does not mediate cell aggregation when overexpressed in *E. coli* laboratory strains^19^, but under certain conditions, it may have a slight indirect effect on these phenotypes^21^. UpaB is composed of an N-terminal signal sequence (residues 1–37), an α-domain (residues 38–500) and a β-domain (residues 501–776) (Fig. 1a). In order to understand the functional properties of UpaB, we cloned, expressed and purified the region encoding the UpaB α-domain (α^UpaB^) from UPEC CFT073 and used analytical ultracentrifugation sedimentation velocity experiments to assess its propensity to self-associate in solution. At 0.5, 1 and 2.2 mg ml^−1^, α^UpaB^ produced a single sedimentation boundary and a continuous sedimentation-coefficient distribution (*c*(*s*)) (Fig. 1b) to give a single species with a standardised sedimentation coefficient of 3.1 s. Analysis by continuous mass distribution (*c*(*M*)) gave a molecular weight of approximately 52.7 kDa, consistent with a monomeric species. Likewise, small-angle X-ray scattering (SAXS) of α^UpaB^ at concentrations <2.7 mg ml^−1^ was consistent with a monodisperse protein population (Supplementary Fig. 1), with the estimated mass, radius of gyration (*R*_g_) and the maximum linear dimension (*D*_max_) from the experimental pair-distance distribution profile (*p*(*r*)) yielding values close to what would be expected for a solution of monomeric α^UpaB^ (Supplementary Table 1). Thus, unlike the α-domain of Ag43 (α^Ag43^), recombinant α^UpaB^ does not self-associate. This fundamental difference in the functional properties of both proteins led us to determine the crystal structure of α^UpaB^.

### Overall UpaB structure

The α^UpaB^ crystal structure was solved to 1.9 Å resolution (crystallographic *R*_factor_ of 17.5%; *R*_free_ 21.8%). The crystallographic refinement statistics are reported in Table 1. The structure was solved from a xenon derivative by single isomorphous replacement with anomalous scattering. One molecule of α^UpaB^ was found in the asymmetric unit. The crystal structure of α^UpaB^ exhibited a right-handed three-stranded β-helix consisting of 13 turns (Fig. 1c), with each triangular turn containing three faces, F1, F2 and F3 faces (Fig. 1d). The β-helix is predominantly stabilised by an inter-strand network of hydrogen bonds. The interior of the β-helix is packed mostly by long stacks of aliphatic residues (Fig. 1e), whereas the exterior is largely acidic in nature. At the C-terminus of the β-helix, α^UpaB^ forms a two-stranded β-sandwich that is capped by a three-stranded β-meander motif. This latter region resembles the autochaperone region that is required for the folding of AT α-domains on the cell surface^22^. A DALI structural alignment revealed that α^UpaB^ shared only a low resemblance to other AT structures in the PDB, with highest similarity to the *Bordetella pertussis* pertactin AT (PDB 1DAB); (18% sequence identity, *Z*-score 24.5 and r.m.s.d of 2.8 Å between 358 equivalent Cα atoms) (Fig. 1f). Compared to α^Ag43^, α^UpaB^ shares only 12% sequence identity, *Z*-score 14.6 and r.m.s.d of 3.8 Å between 242 equivalent Cα atoms (Fig. 1g). Further comparison with other ATs^9–12,17^ revealed the α^UpaB^ β-helix is wider and shorter with a total length of 75 Å. However, the most striking feature of the α^UpaB^ β-helix is the extended β-strands within turns 6–10, which reach up to 32 Å in length. In addition, the strands linking turns 2–3, 3–4, 4–5 and 5–6 are lengthened into a consecutive series of large loops. To date, these structural features have not been observed in the α-domain of any other AT protein^23^.

**Table 1:** UpaB data collection and statistics

	Native	Xenon
Data collection		
Resolution (Å)	50.0–1.97 (2.04–1.97)	48.57–2.50 (2.64–2.50)
Wavelength (Å)	0.9537	1.3776
Space group	*P*3_1_21	*P*3_1_21
Cell dimensions		
* a*, *b*, *c* (Å)	68.6, 68.6, 165.6	69.2, 69.2, 166.0
*α*, *β*, *γ* (degrees)	90.0, 90.0, 120.0	90.0, 90.0, 120.0
Molecules per ASU	1	1
Total no. of reflections	243,523	695,483
No. of unique reflections	32,914 (3219)	16,619 (2351)
Completeness (%)	99.6 (99.6)	100.0 (100.0)
Redundancy	7.4 (6.8)	41.8 (42.9)
* I*/*σ*(*I*)	13.5 (2.7)	30.3 (9.2)
* R*_merge_	7.8 (71.0)	12.3 (56.0)
Phasing		
Resolution		2.5
No. of sites		8
Figure of merit		0.52
Refinement		
Resolution (Å)	33.96–1.97 (2.04–1.97)	
* R*_work_/*R*_free_	17.5/21.8 (0.217/0.245)	
No. of reflections	32,800 (3208)	
No. of atoms		
Protein	3158	
Water	240	
*B*-factors (Å^2^)		
Protein	29.92	
Water	40.13	
RMS deviations		
Bond lengths (Å)	0.007	
Bond angles (degrees)	0.92	

Values in parentheses refer to the highest resolution shell

### UpaB can bind glycosaminoglycans

**Fig. 2:**
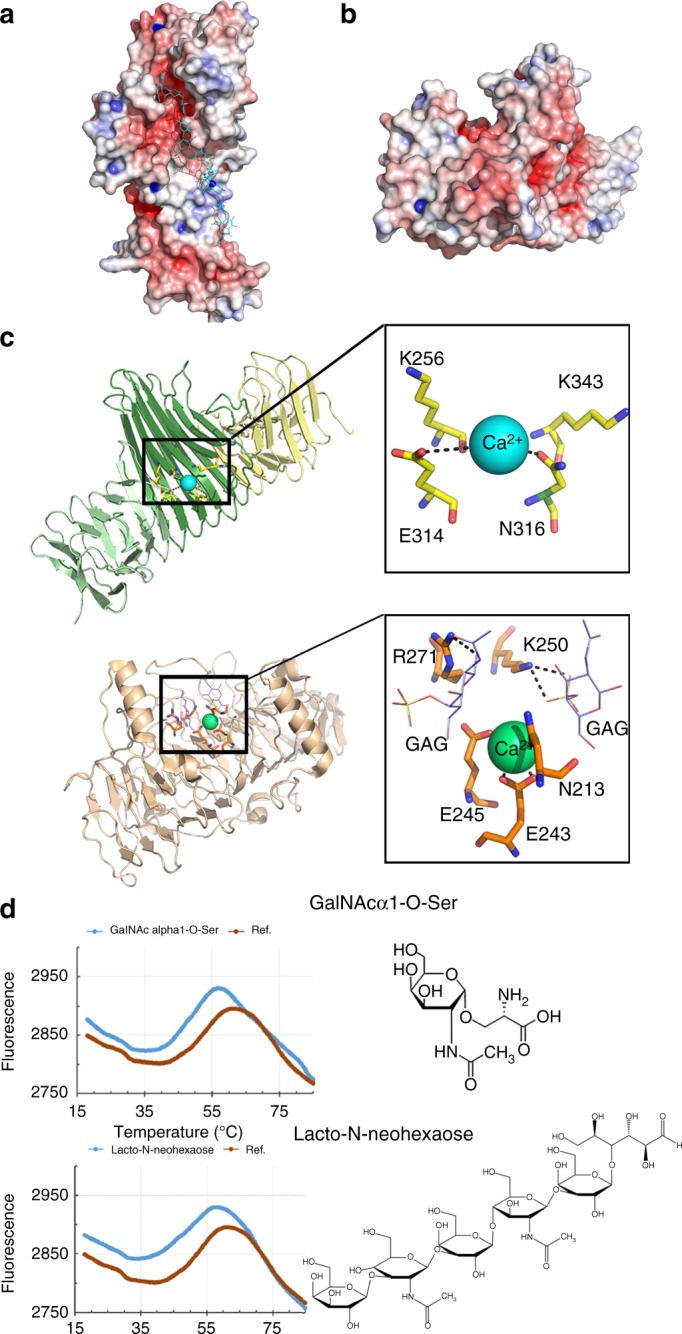
UpaB can bind glycosaminoglycans. Surface representation of **a** α^UpaB^ and **b** top view of α^UpaB^, with electrostatic potential coloured from the most negative (red) to positive (blue), with a range of ± 10 *kT* *e*^−1^. The β-strand extensions contributed by turns 6–10 and long loops protruding from between turns 2–6 form an acidic groove. A GAG was modelled into the α^UpaB^ groove showing that this feature can both accommodate a GAG molecule and place it in proximity to the putative lyase active site. **c** Structural comparison of α^UpaB^ (green) to *P. heparinus* chondroitinase B (wheat; PDB 1DBG). UpaB shares a β-helix structure, groove, bound calcium (cyan and green) and location of a putative lyase active site with chondroitinase B. UpaB has a putative GAG lyase active site (top right panel) consisting of 256 K and 343 K in proximity to a bound calcium (cyan and green) similar to that of chondroitinase B (lower right panel). **d** Melting curve plots showing the fluorescence intensity (relative fluorescence units (RFU)) of Sypro orange as a function of temperature for purified α^UpaB^ in the presence of GalN-α1-O-Ser and Lacto-*N*-neohexaose. The addition of these compounds resulted in a *T*_m_ shift of −3.23 and −3.67 °C, respectively (mean *T*_m_ shift of the 88 carbohydrates screened was <0.7 °C)

The β-strand extensions contributed by turns 6–10 and the long loops protruding between turns 2–6 form a long hydrophilic groove 11 Å wide and 12.5 Å deep on the F1 face of α^UpaB^ (Fig. 2a, b). Sidechains from E165, S188, N189, Q197, T230 and E293 protrude into the groove and largely determine its slightly acidic nature. The results of the DALI search using α^UpaB^ were further analysed to define a role for its groove and revealed that UpaB shared low structural similarity to polysaccharide degrading enzymes (1BHE, 5GKD, 4C2L), which are also composed of a β-helix with a prominent groove for binding polysaccharides^24–26^. The α^UpaB^ groove most closely resembled the glycosaminoglycan (GAG) lyase chondroitinase B (PDB 1OFL) from *Pedobacter heparinus*^27^ (8% sequence identity, *Z*-score 16.5 and r.m.s.d of 3.3 Å) (Fig. 2c). Chondroitinase B is the closest homolog known to interact with human polysaccharides. Importantly, α^UpaB^ shares a putative active site with chondroitinase B and other GAG lyases, located just outside the groove (Fig. 2c). This site comprises UpaB Lys 256 and Lys 343 situated in similar positions to chondroitinase B Lys250/Arg271 Brønsted base/acid pair required to break the glycosidic bonds of GAGs^28^. In chondroitinase B and other GAG lyases, the Lys250/Arg271 would be situated proximal to a bound calcium ion required for neutralisation of the GAG carboxylic group during bond cleavage. Indeed, we identified electron density associated with the α^UpaB^ lysine pair likely to be a bound calcium (Supplementary Fig. 2). Similar to other lyases, this calcium ion would be held in place by the neighbouring α^UpaB^ Glu 314 and Asn 316 residues. The likelihood of a GAG binding within the UpaB groove was tested using docking simulations (Fig. 2a). A model of a GAG was constructed and docked into the α^UpaB^ groove using Autodock Vina. All of the docking conformations showed an interaction with the α^UpaB^ groove, with one of the top conformations displaying an interaction with the putative lyase active site resembling a pre-cleavage state (Supplementary Fig. 2b). This binding conformation exhibited a significant predicted binding affinity of −9.4 kcal mol^−1^ (free energy of binding), based on an extensive hydrogen bonding network between the GAG hydroxyl groups and a number of polar residues within and around the α^UpaB^ groove.

This investigation was followed by the screening of α^UpaB^ against 2788 compounds (including 88 carbohydrate molecules) in a fluorescence thermal shift-based assay (Fig. 2d). Significant binding was shown to two ‘GAG-like’ molecules, Tn Antigen GalN-α1-O-Ser and lacto-*N*-neohexaose (Fig. 2d). GalN-α1-O-Ser closely resembles the *O*-glycosidic-linked saccharide to serine complex that anchors most GAGs to their core proteins, and the lacto-*N*-neohexaose is representative of a main chain GAG^29^. The role of the UpaB groove in this binding was shown by repeating this assay with a UpaB mutant (α^UpaB_G1^), designed by alanine substitutions to the prominent residues that stabilise the GAG interaction identified in our molecular docking studies (E165A, N189A, Q197A, N200A, Q203A, K256A and N316A). Although we confirmed that these alterations did not affect the secondary structure of α^UpaB_G1^ (Supplementary Fig. 3a) and α^UpaB-G1^ behaved in solution similar to the native protein (Supplementary Fig. 3b), this mutant was unable to bind the ‘GAG-like’ molecules as shown by the overlapping melting curve plots of α^UpaB-G1^ in the presence and absence of the GAGs (Supplementary Fig. 3c). Further analyses revealed that α^UpaB^ did not display a broad affinity for some common GAGs found in the urinary tract including chondroitin sulfate A, B, C and heparin sulfate^29,30^ (Supplementary Figs. 4 and 5).

Overall, these results show that α^UpaB^ can bind GAG-like molecules and that this binding is lost when we mutate the GAG-binding site. Our data also indicate that α^UpaB^ may display a considerable substrate specificity (chemical library screening identified only two ‘GAG-like’ molecules out of 2788 compounds and we did not observe binding to four polysaccharides found in the urinary tract) and therefore may interact with a limited range of GAGs that we are yet to identify.

### UpaB contains a FN-binding site

**Fig. 3:**
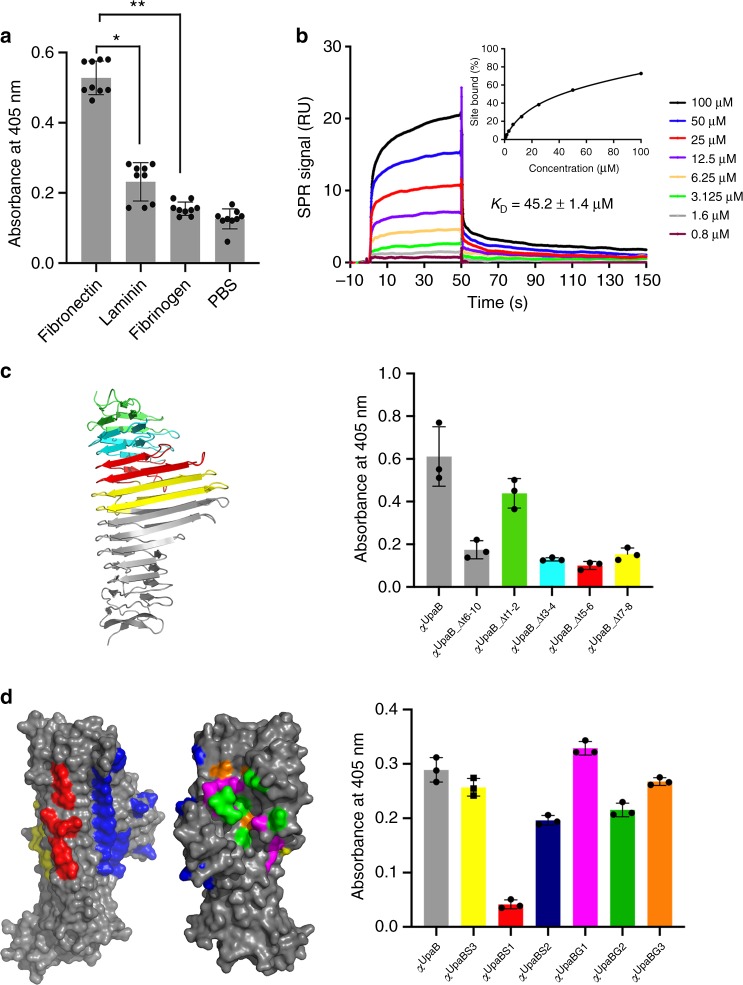
Functional analysis of the UpaB fibronectin-binding site. **a** Assessment of UpaB binding to human fibronectin, laminin and fibrinogen by enzyme-linked immunosorbent assay (ELISA) using a UpaB-specific polyclonal antibody. UpaB showed highest affinity towards fibronectin. Statistical significance was determined by unpaired two-sample *t* test, *P < 0.001, *n* = 9; **P < 0.001, *n* = 9. **b** Surface plasmon resonance analysis of α^UpaB^ binding to immobilised fibronectin. A series of concentrations (0.8–100 µM) of α^UpaB^, as indicated in the sensogram, were injected over fibronectin. The apparent equilibrium dissociation constant *K*_D_ was determined using a steady-state affinity model. The data are expressed as mean ± standard error of the mean (SEM) of three replicates. **c** Assessment of binding to fibronectin by UpaB deletion mutants; α^UpaB-Δt6–10^ (grey); α^UpaB-Δt1–2^ (green), α^UpaB-Δt3–4^ (cyan), α^UpaB-Δt5–6^ (red) and α^UpaB^-^Δt7–8^ (yellow) using ELISA and a fibronectin-specific polyclonal antibody. α^UpaB^ (native) was included as control. Data are shown as the means ± standard deviation of three replicates. **d** Assessment of binding to fibronectin by UpaB mutants containing targeted amino acid substitutions using ELISA and a fibronectin-specific polyclonal antibody. Targeted changes were made to various surface features of UpaB including an acidic patch α^UpaB_S1^ (red; N116A, D119A, N146A, N175A, D217A, K245A, D246A, D281A, R310A and D336A) and polar patch α^UpaB_S2^ (blue; N110A, K111A, N112A, D142A, N171A, D206A, D208A, N212A, N241A, N274A, N276A, N303A, N305A, K325A, D329A, D331A and D349A) on the F2 face, a hydrophobic patch α^UpaB_S3^ (yellow; V151A, I221A, V249A, A252G, A253G, Y285A, Y312A and V339A) between the F2 and F3 faces, along with a hydrophobic α^UpaB_G2^ (green, F101A, Y130A, Y187A, F195A, L201G, L202G, Y260A) and acidic patch α^UpaB_G3^ (orange, E103A, D138A, E165A, E226A) within the GAG binding groove. Binding to fibronectin by α^UpaB_G1^ (E165A, N189A, Q197A, N200A, Q203A, K256A and N316A) was also tested. Alteration of the surface acidic patch S1 abolished the ability of UpaB to bind fibronectin. Data are shown as the mean ± standard deviation of three replicates

In addition to GAG containing proteoglycans, the epithelium is comprised of many other glycoproteins including ECM components^31^. *E. coli* expressing UpaB was previously found to bind these ECM proteins^9^. We examined this further by testing the ability of purified α^UpaB^ to bind human FN, laminin and fibrinogen (Fig. 3a). α^UpaB^ bound strongest to FN, and thus we focussed on understanding this interaction at the molecular level. Using surface plasmon resonance (SPR) we determined a dissociation constant (*K*_D_) of 45.2 ± 1.4 μM between UpaB and FN (Fig. 3b), with the latter immobilised to a CM5 sensor chip using the standard coupling procedure^32^. Although the determined *K*_D_ may be somewhat underestimated owing to the restricted conformational flexibility of the immobilised FN, this binding affinity is consistent with other bacterial FN-binding proteins (including a Fn type III-binding protein)^33–35^. This affinity is also comparable with other physiologically important protein–protein interactions that mediate cell–cell contacts (i.e., T cell receptor–major histocompatibility complex (MHC) complexes range from 2 to 112 μM *K*_D_^36^). Importantly, this binding would not be in the context of a single protein–protein interaction but rather the expression of multiple copies of UpaB on the bacterial cell surface would further enhance the bacterial binding efficiency to FN.

Unlike many other bacterial FN-binding proteins (FnBPs), UpaB does not possess a characteristic GGXXXXV(E/D)(F/I)XX(D/E)T(Xx15)EDT FN-binding repeat (FnBR) sequence^37^. Therefore, to determine the region of α^UpaB^ that binds FN, we generated a series of α^UpaB^ mutants with specific deletions in β-strand turns, followed by overexpression and purification of the corresponding proteins (Supplementary Fig. 6a). Testing of the mutant proteins for their capacity to bind human FN revealed that deletion of the region encompassing the extended β-strands in turns 6–10 (α^UpaB-Δt6–10^) resulted in a significant reduction in binding to FN (Fig. 3c). Further analysis involving the progressive deletion of pairs of β-strand turns from the α^UpaB^ N-terminus through the extended β-strand region, generating α^UpaB-Δt1–2^, α^UpaB-Δt3–4^, α^UpaB-Δt5–6^ and α^UpaB-Δt7–8^, demonstrated that the highest loss in FN binding was caused by deletion of turns 3–8. As such, most of the region encompassing the β-strand extensions comprises the primary site for binding FN. The secondary structure of the deletion mutant that was least affected (α^UpaB-Δt1–2^) and most affected (α^UpaB-Δt5–6^) to binding FN was confirmed by circular dichroism spectroscopy (Supplementary Fig. 3a). Finally, the same mutations were also generated in the full-length UpaB protein, thereby enabling us to assess its capacity to mediate binding to FN upon translocation to the *E. coli* cell surface. These UpaB deletion mutants, when expressed with their β-domain transporter, were all translocated to the cell surface, as confirmed by whole-cell enzyme-linked immunosorbent assay (ELISA) using polyclonal UpaB antibody (Supplementary Fig. 7a). Subsequent whole-cell ELISA experiments revealed that *E. coli* expressing these UpaB mutant proteins bound to FN in a manner consistent with the results obtained using purified recombinant proteins (Supplementary Fig. 7b).

To determine the specific site within turns 3–8 of α^UpaB^ that interacted with FN, we initially investigated the GAG-binding groove on the F1 face, as it was the most prominent structural feature within this region. Utilising our α^UpaB_G1^ GAG-binding mutant, along with other mutants containing amino acid substitutions of hydrophobic (α^UpaB_G2^) and acidic (α^UpaB_G3^) residues within the groove, we found that these regions had no effect on FN binding as determined by ELISA (Fig. 3d). We next examined the other α^UpaB^ faces for possible sites that could bind FN. We made amino acid substitutions to a predominantly acidic patch (α^UpaB_S1^) and polar region (α^UpaB_S2^) on the F2 face and a hydrophobic patch (α^UpaB_S3^) between the F2 and F3 faces (Fig. 3d).

Substitution of residues D116, D119, N146, N175, D217, K245, D246, D281, R310 and D336 on the F2 face to alanine (α^UpaB_S1^) caused almost complete loss of FN binding as determined by ELISA, while maintaining the correct secondary structure of α^UpaB_S1^ based on circular dichroism spectroscopic analysis (Supplementary Fig. 3a) and displaying a behaviour in solution similar that of the native protein (Supplementary Fig. 3b). This result mapped the FN-binding site to a ladder of charged/polar residues that are contributed from β-strands or loops in consecutive rungs of the α^UpaB^ β-helix. The only established mode of interaction between FnBPs and FN involves the donation of a series of structurally disordered FnBPs to FN, which upon binding each form an additional β-strand within type I FN modules^37,38^. Thus the α^UpaB^–FN interaction is unique, with interacting residues contributed from already formed β-strands held tightly within the α^UpaB^ β-helix by a hydrogen-bonding network.

### UpaB can bind FN type III

**Fig. 4:**
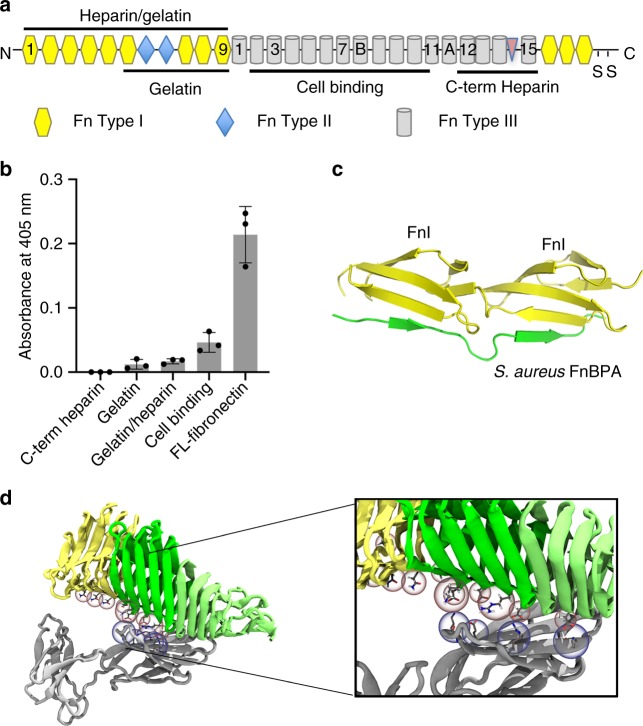
UpaB binds fibronectin type III. **a** Fibronectin domain organisation composed of 12 type I modules (FnI), 2 type II modules (FnII) and 15–17 type III modules (FnIII). Commercially available fragments used in this work include the heparin/gelatin FnI_1–9_, FnII_1–2_, the gelatin FnI_6–9_, FnII_1–2_, the cell binding FnIII_2–11_ and the C-terminal heparin FnIII_12–15_ fragments. **b** Binding of fibronectin fragments, as well as full-length (FL) fibronectin, to UpaB measured by enzyme-linked immunosorbent assay using an UpaB-specific polyclonal antibody. Data are shown as the mean ± standard deviation of three replicates. **c** Tandem β-zipper interaction between Fn binding repeat peptides from *S. aureus* Fn-binding protein A (FnBPA) and Fn type I modules 2 and 3. The established mode of interaction between bacterial proteins and fibronectin involves the donation of up to 11 structurally disordered bacterial fibronectin repeats to form additional β-strands with consecutive FnI modules. **d** Model of the UpaB-FnIII interaction derived from NAMD simulations using the structures of UpaB and the FnIII_1–2_ fragment (PDB: 2HA1), showing predominately hydrogen bonding between charged residues of UpaB (in particular, D246, D310, D336 and D375) and FnIII_1_ (residues K32, R36, K40 and E70). The equivalent mutant simulation did not show any appreciable hydrogen bonding

In order to further investigate the atypical mode of interaction between UpaB and FN, we determined the region of FN bound by α^UpaB^. FN is composed of 12 type I modules (FnI), 2 type II modules (FnII) and 15–17 type III modules (FnIII)^38^. We obtained commercially available fragments of human FN which included a 45 kDa gelatin-binding fragment (FnI_6–9_, FnII_1–2_), a 70 kDa heparin/gelatin-binding fragment (FnI_1–9_, FnII_1–2_), a 120 kDa cell-binding fragment (FnIII_2–11_) and a 40 kDa C-terminal heparin-binding fragment (FnIII_12–15_)^32^ (Fig. 4a, Supplementary Fig. 6b). The binding of α^UpaB^ to these FN fragments determined by ELISA revealed that it displayed strongest interaction with the cell binding fragment (FnIII_2–11_) and weak binding to the gelatin (FnI_6–9_, FnII_1–2_) and heparin/gelatin (FnI_1–9_, FnII_1–2_) fragments (Fig. 4b). Given the size of UpaB, this maps its binding site on FN to the first FnIII units in the cell-binding fragment, possibly also including some interaction with the neighbouring FnI units in the gelatin-binding fragment (note that the gelatin [FnI_6–9_, FnII_1–2_] and heparin/gelatin [FnI_1–9_, FnII_1–2_] fragments overlap in this region). The difference observed between UpaB binding to full-length FN compared to its fragments could be attributed to the lack of the FnIII_1_ unit within any of the commercially available fragments, which given its location would be an important contribution to the UpaB–Fn interaction. FnIII_1–2_ are valid targets for bacterial pathogens such as UPEC, as these 2 units are known to be involved in FN matrix assembly, whereby their disruption could facilitate bacterial spread^39^.

Bacterial interactions with FnIII are uncommon; most of the 100 known bacterial FnBPs bind to the FnI heparin- and gelatin-binding domains via a β-zipper interaction^38^ (Fig. 4c). However, there are an increasing number of bacterial proteins including *Haemophilus influenzae* Hap^40^, *Staphylococcus epidermidis* Embp^41^, *Salmonella enterica* serovar Typhimurium ShdA^42^ and *Pasteurella multocida* PM1665^32^ that bind to FnIII. The AT Hap (13% sequence identity) also primarily binds to FnIII_1–2_ via four small binding motifs. These motifs are absent in UpaB and both the UpaB and Hap FN-binding sites do not share the same location.

Overall, the structural basis for how bacterial proteins interact with the FnIII fragment of FN has not been determined, and thus we investigated this interaction using molecular dynamics simulations with our α^UpaB^ crystal structure and the structure of human FnIII_1–2_ (2HA1)^39^ (Fig. 4d; Supplementary Movie 1). To visualise this interaction, we also ran simulations with the α^UpaB_S1^ mutant that had lost its capacity to bind FN (Supplementary Movie 2). Modelling simulations were performed using NAMD 2.12^43^ for a cumulative total of 1.2 μs for each system (3 replicates of 400 ns each). Though the simulations are too short in timescale terms of protein–protein interactions to demonstrate specific binding, they do provide plausible binding mechanisms, showing that α^UpaB^ could interact with FnIII via complementary charged residues without unfolding and/or donating β-strands. Specifically, our α^UpaB^-FnIII_1–2_ simulations indicate that α^UpaB^ primarily interacts with FnIII_1_, through hydrogen bond interactions mediated by α^UpaB^ D246, R310, D336 and D375 residues with complementary charged areas on FnIII, particularly residues K32, K40, E70, R36 and of FnIII_1_. Substitutions of the α^UpaB^ FnIII-interacting residues to alanine in the α^UpaB_S1^ mutant greatly reduced hydrogen bond interactions observed in the simulations (Supplementary Movie 2). β-Helix–β-helix interactions have been observed previously for Ag43 and some trimeric ATs^17,44,45^. However, these studies show how an AT β-helix can interact with another type of fold, namely the β-sandwich fold of FnIII.

### UpaB is highly conserved and immunogenic during UPEC infection

To determine whether the structural features of UpaB required for binding FnIII and GAGs are conserved across the *E. coli* species, we screened the NCBI public database and an *in-house* collection, which were represented by 2818 draft and 199 complete *E. coli* genome sequences. Overall, the *upaB* gene was present in 1019 strains in this collection (34%) and was found in UPEC strains as well as intestinal pathogenic, commensal and other extra-intestinal pathogenic strains. Analysis of these 1019 translated UpaB amino acid sequences revealed that 95% (968/1019) shared an amino acid sequence identity >89%. Comparison of the translated UpaB amino acid sequence from seven completely sequenced UPEC strains showed that the putative GAG lyase active site was strictly conserved; there was also high conservation of the residues that contribute to the acidic groove, as well as the residues that interact with FnIII (Supplementary Fig. 8). We also examined whether an immunological response against UpaB was elicited during human UPEC infection and showed that plasma samples from urosepsis patients infected with UpaB-positive *E. coli* strains produced significantly higher anti-α^UpaB^ antibody titres compared to healthy individuals (Supplementary Fig. 9).

### Relevance of GAG and FN-binding regions in vivo

**Fig. 5:**
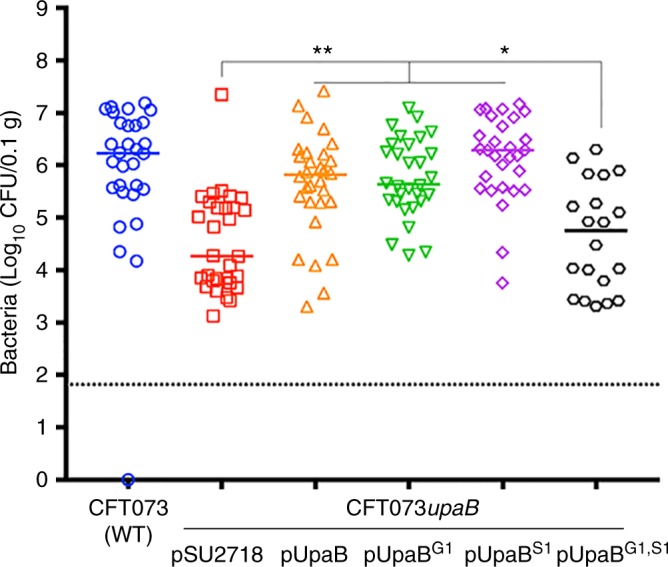
UPEC colonisation of the mouse bladder is enhanced by UpaB GAG- and fibronectin-binding interactions. C57BL/6 mice were challenged transurethally with wild-type CFT073, CFT073*upaB*(pSU2718), CFT073*upaB*(pUpaB), CFT073*upaB*(pUpaB^G1^), CFT073*upaB*(pUpaB^S1^) and CFT073*upaB*(pUpaB^G1, S1^). The results represent log_10_ CFU/0.1 g bladder tissue of individual mice at 24 h post-infection, and the horizontal bars mark group medians. A minimum of 20 mice were assessed per group (pooled from at least 2 independent experiments). Data were compared using Kruskal–Wallis analysis of variance (ANOVA) with Dunn’s multiple comparisons correction (*P < 0.05; **P < 0.01)

We previously showed that mutation of *upaB* in CFT073 led to decreased bladder colonisation in experimental mice^19^. In order to examine how the GAG- and FN-binding properties of UpaB impact its function in vivo, we constructed plasmids containing the S1, G1 and double S1–G1 mutations in the full-length *upaB* gene. These plasmids were transformed into our *upaB* mutant strain (CFT073*upaB*) to generate a set of strains with plasmid pSU2718 (vector control), pUpaB (wild-type (WT) UpaB), pUpaB^G1^ (UpaB with mutated GAG-binding site), pUpaB^S1^ (UpaB with mutated FN-binding site) or pUpaB^G1, S1^ (UpaB with mutated GAG- and FN-binding sites). Next, we examined the capacity of our CFT073*upaB* complemented strains to colonise the mouse bladder. In these experiments, CFT073*upaB* complemented with pUpaB, pUpaB^G1^ and pUpaB^S1^ restored bladder colonisation at 24 h post-infection to a level equivalent to colonisation by WT CFT073 (Fig. 5). In contrast, complementation with either the vector control plasmid pSU2718 or pUpaB^G1, S1^ did not restore bladder colonisation to WT levels, and these levels were significantly reduced at 24 h post-infection compared to colonisation by CFT073*upaB* containing pUpaB, pUpaB^G1^ or pUpaB^S1^ (Fig. 5). This lack of complementation by pUpaB^G1, S1^ was not due to lack of expression of the mutant protein on the cell surface, as demonstrated by western blot analysis and whole-cell ELISA (Supplementary Fig. 10a, b). The stability of the pUpaB^G1,S1^ mutant was also confirmed by purification and biophysical characterisation of recombinant α^UpaB_G1,S1^ (Supplementary Fig. 3a and Supplementary Fig. 10c). A similar colonisation profile was observed for each of the UPEC strains in the urine of these experimentally infected mice (Supplementary Fig. 10d).

## Discussion

AT adhesins are a common group of proteins that play a central role in bacterial pathogenesis. They allow bacteria to adhere to human cells, aggregate with other bacteria and form biofilms, all key facilitators of bacterial virulence. Currently, our understanding of the function of ATs is limited, and detailed information at the level of atomic structure as well as the precise molecular mechanisms that govern their interaction with target molecules is lacking. Here we describe the structure and mechanism of action for UpaB, a recently described AT adhesin from UPEC.

UpaB differs from other characterised AIDA-I AT adhesins as it does not self-associate. Self-association of AIDA-I ATs on the bacterial cell surface is the mechanism by which AIDA-I ATs promote bacterial aggregation and biofilm formation^17^. The lack of this feature in UpaB is consistent with previous studies which showed that UpaB overexpression did not impact on these phenotypes^19,46^. Subsequent determination of the α^UpaB^ structure revealed an unprecedented departure from the common β-helix fold previously determined for all other ATs and led to the definition of several novel features in the architecture of UpaB. Extensions of β-strands within α^UpaB^ together with large loops create a long acidic groove that can bind GAG. This site bears structural similarity to characterised GAG lyases^28^ and promoted the binding of UpaB to GAG-‘like’ substrates, thus supporting a potential polysaccharide-binding site in an AT protein.

We also show that UpaB can bind to human FN. Using a series of UpaB mutants in combination with different fragments of FN, we showed that this occurs via interaction with type III FN, most likely at FnIII_1–2_. Despite human FN being one of the most common targets for bacterial adhesins, a detailed mechanistic description of the structural basis for binding to FnIII is lacking^32,38,40–42^. In UpaB, this interaction involves the folded UpaB β-helix presenting a ladder of charged/polar residues that interact with complementary charges on FnIII. This is in contrast to previously characterised FnBP–FN interactions, which occur through the donation of a series of disordered repeats from the FnBP to form a tandem β-zipper interaction with consecutive FnI domains^37,47^. This mode of UpaB binding to FnIII might also be utilised by other bacterial proteins that bind FnIII. Indeed, binding interactions involving β-helices have been increasingly observed among ATs and other proteins^17,44,45^.

Our previous work showed that deletion of *upaB* reduces UPEC colonisation of the mouse bladder^19^. The in vivo data we present here show that mutating either the GAG-binding site or the FN-binding site does not affect UPEC colonisation, while mutating both binding sites leads to a modest decrease in colonisation. One possible interpretation of these results would be that UPEC colonisation may be facilitated by UpaB binding to both GAG and FN. However, it is possible that the multiple mutations required to inactivate both binding domains may have altered the stability or other functional features of the UpaB protein. Therefore, further work is needed to clarify the potential relevance of UpaB binding to GAG and FN during infection.

## Methods

### Bacteria and growth conditions

*E. coli* strains and plasmids used in this study are listed in Supplementary Table 2. Bacteria were routinely grown at 37 °C on solid or in liquid Luria-Bertani (LB) medium supplemented ampicillin (100 μg ml^−1^) or chloramphenicol (30 μg ml^−1^).

### Cloning, expression and purification of α^UpaB^

The coding sequence for the *upaB* alpha domain (residues 38–500, locus tag c0426) was PCR-amplified from UPEC CFT073 genomic DNA using primers 4326-UpaB-F and 4327-UpaB-R containing ligation-independent cloning (LIC) overhangs (Supplementary Table 2). Using LIC cloning, the amplified gene was inserted into a modified version of a pMCSG7^17^ vector, which encodes a N-terminal his_6_-tag followed by a thioredoxin (TRX) domain and a TEV protease cleavage site. The resulting plasmid, pUpaBα, was used for the expression of α^UpaB^ and introduces three residues at the N-terminus (i.e. SNA) upon removal of the his_6_-TRX-tag with TEV. The α^UpaB^ protein was expressed in *E. coli* BL21 (DE3) pLysS cells (Invitrogen) using autoinduction (24 h at 30 °C) in the presence of the appropriate antibiotics (ampicillin 100 μg ml^−1^, chloramphenicol 34 μg ml^−1^). Cells were harvested, resuspended in 25 mM Tris pH 7.5 and 150 mM NaCl and lysed by cell sonication. The lysate was cleared by centrifugation and loaded onto a HisTrap column (GE Healthcare). Proteins were eluted in a gradient of 0–500 mM imidazole. Fractions containing α^UpaB^ were cleaved with TEV protease and the uncleaved protein was removed by further nickel affinity chromatography. Size exclusion chromatography (Superdex S-75 GE Healthcare) in 25 mM Hepes and 150 mM NaCl pH 7.0, was used to further purify α^UpaB^ as assessed by sodium dodecyl sulfate-polyacrylamide gel electrophoresis (SDS-PAGE).

The pα^UpaB^ plasmid was used as the parent vector for the construction of all α^UpaB^ mutants, namely α^UpaB-Δt6–10^, α^UpaB-Δt1–2^, α^UpaB-Δt3–4^, α^UpaB-Δt5–6^, α^UpaB-Δt7–8^, α^UpaB-G1^, α^UpaB-G2^, α^UpaB-G3^, α^UpaB-S1^, α^UpaB-S2^, α^UpaB-S3^ and α^UpaB-G1,S1^ (Supplementary Table 2). All constructs were generated by Epoch Life Science, confirmed by sequencing and transformed into *E. coli* BL21 DE3 pLysS (Supplementary Table 2). The α^UpaB^ mutants were expressed and purified as described for the native α^UpaB^.

### Crystallisation

Crystals of α^UpaB^ were grown at 20 °C using the hanging-drop vapour-diffusion technique. Crystals grew at 20 mg ml^−1^ in 0.1 M sodium acetate pH 4.8, 0.2 M ammonium sulfate and 28% (w/v) PEG 4000. Crystals pre-equilibrated in reservoir solution containing 20% glycerol were flash-cooled in liquid nitrogen. Xenon derivatisation was performed using a Xenon chamber (Hampton Research) at 20 bar for 1 min before flash freezing.

### Structure determination and refinement

Native data were collected (*λ* = 0.954, −163 °C) from a single crystal with an ADSC Q315r CCD detector on the MX2 micro-crystallography beamline at the Australian Synchrotron. The data were integrated and scaled with HKL2000^48^. Anomalous data were collected (*λ* = 1.3776, −163 °C) from 2 crystals at the MX2 beamline. This data was integrated, scaled and merged using XDS/XSCALE^49^. All crystals belonged to spacegroup *P*3_1_21 with similar cell dimensions of *a* ≈ 69 Å, *b* ≈ 69 Å, *c* ≈ 166 Å and *α* = 90.0°, *β* = 90.0° and *γ* = 120.0°. This was consistent with one α^UpaB^ molecule per asymmetric unit. The structure of α^UpaB^ was determined by single isomorphous replacement using anomalous signal from Xenon. SHELX C,D,E^50^ was used to find the Xenon atoms, phasing and density modification. Eight Xenon atoms were found per asymmetric unit. ARP/wARP^51^ was used for initial model building against the experimental phases. This model underwent rounds of manual model building using the program COOT^52^ and refinement using Refmac5^53^ and phenix.refine^54^ to 1.97 Å using native data. The quality of the model was monitored during refinement by the Rfree value, which represented 5% of the data. The structure was validated by the MolProbity^55^ server and the figures were created with PyMOL^56^. Ramachandran statistics showed 97.87% of residues in the most favoured region and 2.13% in the allowed regions. Details of data-processing statistics and final refinement values are summarised in Table 1.

### Analytical ultracentrifugation

Sedimentation velocity experiments were performed in a Beckman Coulter model XL-I analytical ultracentrifuge with a An50-Ti rotor. Double-sector quartz cells were loaded with 400 μl of buffer (25 mM Tris pH 7.5 and 150 mM NaCl) and 380 μl α^UpaB^ at 0.5, 1 and 2.2 mg ml^−1^. Initial scans were performed at 725 × *g* to determine the optimal wavelength and radial positions. Absorbance readings were collected at 280 nm and 128,794 × *g*. at 20 °C. Solvent density, solvent viscosity and estimates of the partial specific volume of α^UpaB^ (0.7203 ml g^−1^) at 20 °C were calculated with SEDNTERP^57^. Data were analysed using *c*(*s*) and *c*(*M*) with SEDFIT^58^.

### SAXS data collection and analysis

Data were collected on the SAXS–WAXS beamline at the Australian Synchrotron. Serial dilutions of a 2.7 mg ml^−1^ stock were made to give samples with concentrations between ~0.1 and 2.7 mg ml^−1^. All samples were centrifuged at 10,000 × *g* prior to being loaded into a 96-well plate. To minimise the effects of radiation damage, samples (~80 μl) were maintained at 283 K and drawn into a capillary from the 96-well plate and flowed past the beam. All measured two-dimensional data were averaged and corrected for transmission, solvent scattering and detector sensitivity and radially averaged to produce I(*q*) vs. *q* profiles using Scatterbrain (v 2.7.1).

The estimated molecular masses were calculated using values for contrast and partial specific volume predicted from the protein sequence using MULCh (v 1.1)^59^ along with the Porod volume. Data processing and Guinier analysis was performed using Primus (v 3.2)^60^. The pair-distance distribution function, *p*(*r*), was generated from the experimental data using *GNOM* (v 4.6)^61^, from which *I*(0), *R*_g_ and *D*_max_ were determined.

The program *CORAL* (v 1.1)^62^ was used to generate 16 rigid-body models of the protein, where the missing N- and C-terminal residues from the crystal structure (PDB: 6BEA) were modelled as dummy residues. All models were qualitatively similar, and the model with the lowest *χ*^2^ was chosen as the representative structure. The program *DAMMIN* (v 5.3)^63^ was used to generate 16 molecular envelopes, which were averaged and filtered using the program *DAMAVER* (v 2.8)^64^. The SAXS data and models have been deposited in the SASBDB^65^.

### Polysaccharide lyase assay and gel

Polysaccharide lyase assays were performed with human chondroitin sulfate A/C (Sigma), human chondroitin sulfate B (Sigma) or human heparin sulfate (Sigma) at 0.5 mg ml^−1^ in 100 mM Tris 50 mM sodium acetate pH 8.0 and 10 mM CaCl_2_ to final volume of 100 μl. Purified α^UpaB^ was added to a final concentration of 0.05 mg ml^−1^ along with the negative control Antigen 43, with the positive control chondroitin lyase ABC (Sigma) used at 0.005 mg ml^−1^. Assays were set-up in 96-well microplates (UV-Star Greiner) and substrate cleavage was followed by A_232_ measurements every 4 min for 2 h at 37 °C, using an EnSpire 2300 multilabel reader (Perkin Elmer). Assay samples were then analysed by SDS-PAGE (4–12%) with Alician Blue/Silver staining.

### ELISA of glycosaminoglycans, FN, laminin or fibrinogen with α^UpaB^

ELISAs of the glycosaminoglycans human chondroitin sulfate A, B, C and heparin sulfate were performed by coating the molecules onto Nunc Maxisorp flat-bottom 96-well plates (Thermo Scientific) at 5, 10, 20 and 40 μg ml^−1^. Plates were blocked with 1% w/v bovine serum albumin and probed with 10 µg ml^−1^ of α^UpaB^. The binding of α^UpaB^ was detected using a UpaB-specific polyclonal antibody (1 in 500 dilution in phosphate-buffered saline (PBS))^19^ followed by alkaline phosphate-conjugated goat anti-rabbit IgG (1 in 10,000 dilution in PBS (Sigma A3687)). For human laminin (10 µg ml^−1^, Sigma), human fibrinogen (10 µg ml^−1^, Sigma) and full-length human FN (10 µg ml^−1^, Sigma), ELISAs were performed in the same manner. To detect binding to immobilised UpaB or UpaB mutants, ELISA plates were coated with purified α^UpaB^, truncates or α^UpaB^ surface/groove mutants (10 µg ml^−1^) and probed with 10 µg ml^−1^ each of full-length human FN or FN fragments; including the gelatin-binding (Sigma), heparin/gelatin (Sigma), cell-binding (Merck Millipore) or C-terminal heparin-binding (Merck Millipore) α-chymotryptic FN fragments. The binding of FN and FN fragments was detected using anti-FN antibody (1 in 1000 dilution in PBS (Sigma F3648)) followed by alkaline phosphate-conjugated goat anti-rabbit IgG (1 in 10,000 dilution in PBS (Sigma A3687)). The reaction was developed in the presence of alkaline phosphatase substrate (Sigma) and absorbance was read at 405 nm.

### Fluorescence thermal shift assays

Fluorescence thermal shift assay was conducted in 384-well plate format with an assay volume of 10 µl. Recombinant α^UpaB^ protein sample and 5000× Sypro-Orange (Invitrogen) were diluted and mixed in a Hepes buffer (20 mM HEPES, 150 mM NaCl, pH 7.5) to a protein concentration of 0.5 µg per well and 5× Sypro-Orange. Because of the low signal intensity of α^UpaB^, higher than usual protein concentration was used. After adding the protein Sypro-Orange mix to a PCR plate, testing compounds were added using a Labcyte Echo550 acoustic liquid transfer robot. Plates were mixed, sealed with optical clear plastic seal and centrifuged. Thermal scanning coupled with fluorescence detection was performed on a CFX384 qPCR machine at 1.5 °C min^−1^ from 10 °C to 85 °C. Data analysis was performed using the in-house software excelFTS, which uses IDBS XLfit for fitting the fluorescence data to a Boltzmann function to determine the melting temperature *T*_m_ and other thermal transition parameters. Two compound libraries of 2700 molecules and 88 molecules each were screened with α^UpaB^. No hit compounds were found from the 2700 molecule Spectrum collection of known and experimental drugs and natural products (MicroSource Discovery). The screen of 88 carbohydrates yielded two hits.

### Circular dichroism spectroscopy

An Aviv model 420 Circular Dichroism Spectrometer was used to investigate the structural properties of α^UpaB^ proteins at 0.3 mg ml^−1^ in 25 mM Hepes pH 7.0 and 150 mM NaCl. Wavelength scans were performed with 0.5 nm steps between 200 to 250 nm at 20 °C.

### ELISA of whole cells expressing UpaB with FN

Full-length *upaB* from UPEC CFT073 was cloned into plasmid pSU2718 at *Xba*I-*Hind*III restriction sites with primers 6460-UpaBF1 and 6461-UpaBR (Supplementary Table 2). The resultant parent plasmid (pUpaB) was used for construction of all UpaB deletion mutants used in this study (Supplementary Table 2). Specific mutations and deletions were introduced into *upaB* by Epoch Life Science to generate the following plasmids [pUpaB^*G1*^, pUpaB^*S1*^, pUpaB^*Δt1–2*^, pUpaB^*Δt3–4*^, pUpaB^*Δt5–6*^ and pUpaB^*Δt7–8*^]. All constructs were confirmed by sequencing and transformed into MS427 (Supplementary Table 2). For all assays, overnight cultures of bacterial cells were normalised to an optical density at 600 nm (OD_600 nm_) of 1.0. Whole-cell ELISAs were performed as described for purified UpaB proteins. Whole-cell ELISAs for the detection of UpaB and UpaB deletion mutants on the *E. coli* cell surface were performed using polyclonal anti-α^UpaB^ antibody (1 in 500 dilution in PBS) and detected using alkaline phosphate-conjugated goat anti-rabbit IgG (1 in 10,000 dilution in PBS (Sigma A3687)). The interaction of UpaB deletion mutants with FN was examined by whole-cell ELISA using anti-FN antibody (1 in 1000 dilution in PBS (Sigma F3648)) and detected using alkaline phosphate-conjugated goat anti-rabbit IgG (1 in 10,000 dilution in PBS (Sigma A3687)).

### SPR measurements

A Biacore T200 biosensor instrument was used to measure the affinity of the interaction of UpaB with full-length human FN. FN was covalently immobilised onto a CM5 chip at two different densities, 1000 RU and 5000 RU, using amine coupling method. SPR experiments were performed at 25 °C using PBS-T (1× PBS pH 7.4 and 0.05% Tween 20) as the running buffer. To generate binding data, UpaB at concentrations ranging from 100 to 0.8 µM was injected over immobilised FN at a constant flow rate of 90 ml min^−1^ for 50 s; UpaB dissociation was monitored by flowing running buffer at 90 ml min^−1^ for 150 s. The surface was regenerated after each cycle by injecting 0.1% SDS. Steady-state equilibrium analysis was carried out using the Biacore T200 evaluation software. *K*_D_ is expressed as mean ± standard error of the mean (SEM). Experiments were conducted on three independent occasions with fresh immobilisation.

### Mouse infections

Plasmids containing the full-length *upaB* gene with mutations in the GAG-binding site (pUpaB^G1^), FN-binding site (pUpaB^S1^) and both binding sites (pUpaB^G1, S1^) were generated by Epoch Life Science. These plasmids, together with a plasmid containing the full-length *upaB* gene (pUpaB) and the vector control (pSU2718), were transformed into CFT073*upaB*, respectively, to generate the strains used in the mouse UTI model. Female C57BL/6 mice aged 10–12 weeks (Animal Resources Centre) were inoculated by transurethral infection to deliver approximately 10^8^ bacteria to the bladder, as described elsewhere^66^. Briefly, mice (*n* = 10 per group) were anaesthetised by inhalation exposure to isoflurane and a sterile Teflon-coated catheter attached to a 1 ml syringe was used to deliver 50 µl of PBS containing ~10^8^ colony-forming units (c.f.u.) of bacteria (at a rate of 5 µl s^−1^) to the bladder. Urine samples were collected 24 h after challenge and were diluted in PBS and plated on LB agar or LB agar supplemented with 30 µg ml^−1^ chloramphenicol, as appropriate, for colony counts. Subsequently, the mice were euthanised (using isoflurane overdose followed by cervical dislocation), and the bladders and kidneys were collected, weighed and homogenised in sterile PBS. The tissue homogenates were diluted and plated on agar as above to quantify c.f.u. per 0.1 g tissue. Data are compiled from at least two independent experiments.

### ELISA to detect anti-α^UpaB^ antibodies from urosepsis patients

Blood plasma was collected from 45 patients presenting with urosepsis at the Princess Alexandra Hospital (Brisbane, Australia) along with matching blood culture UPEC isolates. Isolates were screened for the presence of *upaB*, resulting in 33 plasma samples with matching infecting strains positive for *upaB* to be assessed. Forty-two plasma samples from healthy volunteers were obtained as controls. Recombinant UpaB α-domain (αUpaB; 10 µg ml^−1^) was coated onto Nunc Maxisorp flat-bottom 96-well plates (Thermo Scientific), plasma samples were added and peroxidase-conjugated anti-human IgG (1:30,000 dilution in 5% skim milk (Sigma A0170)) was applied as a secondary antibody for detection (incubated at 37 °C for 90 min). Plates were developed with 3,3’,5,5’-tetramethylbenzidine with absorbance determined using a SpectraMax 190 Absorbance Microplate Reader at 450 nm. Statistical analysis between patient and healthy plasma was performed using an unpaired two-sample *t* test.

### Sequence analysis

The *upaB* sequences from the NCBI database and *in house* collection were compared and aligned using CLC Main Workbench (Qiagen).

### Modelling

Models for dynamics simulations were constructed for both α^UpaB^ and α^UpaB_S1^ using the UpaB crystal structure and the FnIII_1–2_ crystal structure (2HA1). UpaB models were positioned approximately 10 Å from the FN fragment and solvated with TIP3P and electrically neutralised with 0.15 M sodium chloride. The models initial dimensions of 108 × 81 × 120 Å, contained 100,010 and 100,015, atoms respectively. Each model was run independently 3 times for 400 ns, performed with NAMD2.12 34, for a cumulative total of 2.4 µs of simulation. Simulations were performed with NAMD2.12^43^. Long-range Coulomb forces were computed with the Particle Mesh Ewald method with a grid spacing of 1 Å. Two-fs timesteps were used with non-bonded interactions calculated every 2 fs and full electrostatics every 4 fs while hydrogens were constrained with the SHAKE algorithm. The cut-off distance was 12 Å with a switching distance of 10 Å and a pair-list distance of 14 Å. The temperature was set to 310 K. Pressure was controlled to 1 atmosphere using the Nosé–Hoover Langevin piston method employing a piston period of 100 fs and a piston decay of 50 fs. Trajectory frames were captured every 100 ps. Simulation trajectories were viewed and mapped with VMD^67^.

Docking of the GAG into UpaB utilised a model based on the NMR structure of unsulfated chrondroitin (PDB: 2KQO). Docking of the GAG model was performed against the UpaB structure using Autodock Vina^68^. A 52 × 24 × 46 Å search space was set up to cover the UpaB surface and groove. Standard chemical bond torsions were applied to the GAG molecule and UpaB was kept rigid apart from the following residues: N189, Q197, K256, and K343. Docking conformations were ranked against the predicted free energy of binding (kcal mol^−1^).

### Ethics statement

All animal experimentation was conducted in accordance with the guidelines of the National Health and Medical Research Council. The Griffith University Animal Ethics Committee approved this study (MSC/01/18/AEC). The use of human blood plasma from patients was approved by the institutional review board of the Princess Alexandra Hospital (2008/264). The need for patient informed consent was waived, as the primary purpose for the collection of these samples was for other diagnostic procedures, and all patient information was de-identified. The collection of human blood from control subjects was approved by the institutional review board of Griffith University (MSC/18/10/HREC). Informed consent was obtained from all control subjects.

### Reporting summary

Further information on research design is available in the Nature Research Reporting Summary linked to this article.

## Supplementary information

Supplementary Information

Description of Additional Supplementary Files

Supplementary Movie 1

Supplementary Movie 2

Reporting Summary

## Data Availability

Coordinates and structure-factor files have been deposited in the Protein Data Bank, with accession code 6BEA. Scattering data and models have been deposited in the SASBDB with accession code SASDC45.
