# Supplementary material for: Unique structural features of a bacterial autotransporter adhesin suggest mechanisms for interaction with host macromolecules
Source: Nat Commun. 2019 Apr 29;10:1967. doi: 10.1038/s41467-019-09814-6 (PMC6488583; doi:10.1038/s41467-019-09814-6)
Supplement: Supplementary file 1 — Supplementary Information [file 41467_2019_9814_MOESM1_ESM.pdf]

**Supplementary information**

# UNIQUE STRUCTURAL FEATURES OF A BACTERIAL AUTOTRANSPORTER ADHESIN SUGGEST MECHANISMS FOR INTERACTION WITH HOST MACROMOLECULES

Paxman *et al.*

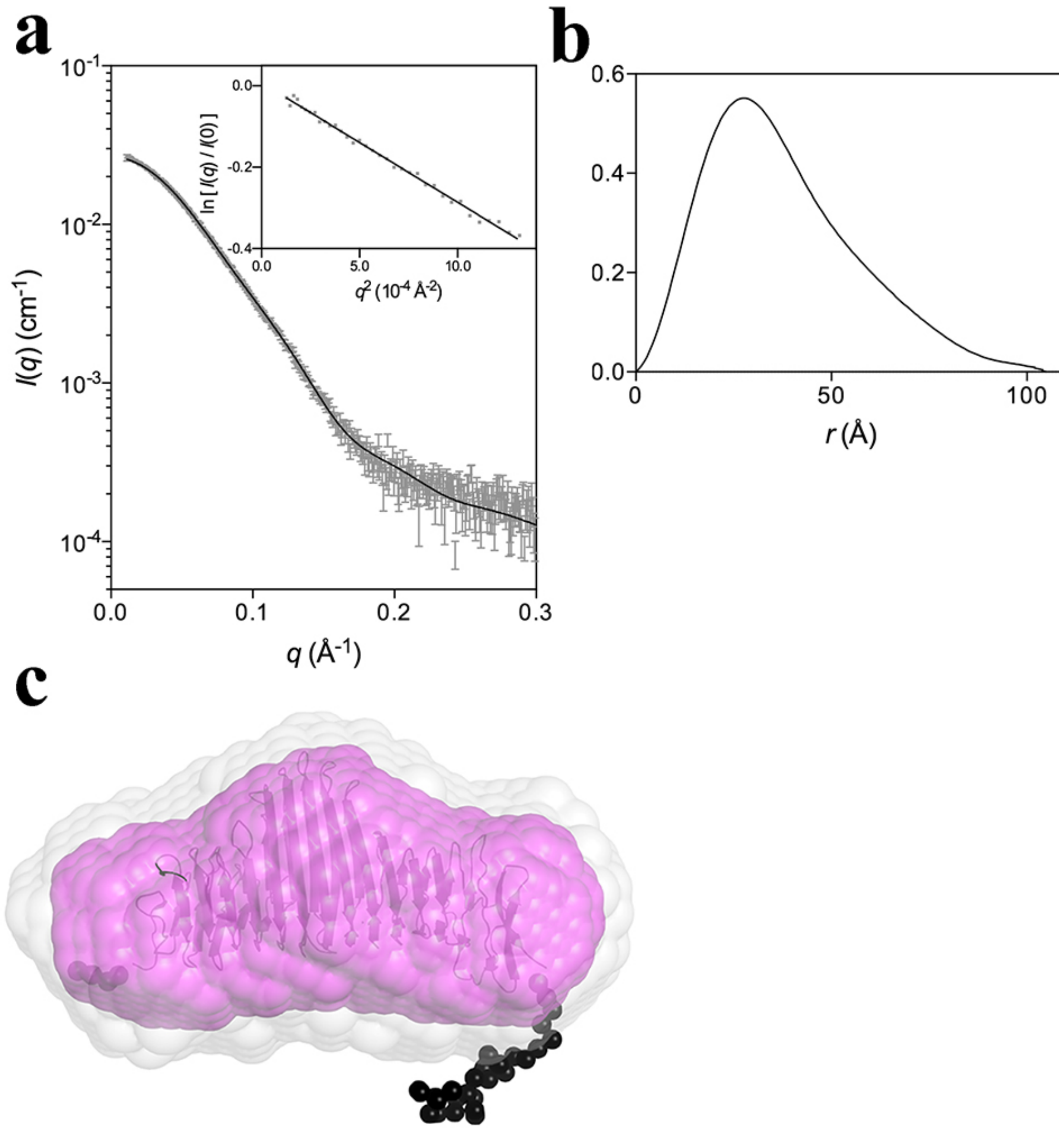


**Supplementary Figure 1:** Small-angle X-ray scattering from $\alpha^{UpaB}$. **(a)** The measured scattering data for $\alpha^{UpaB}$ (grey). The predicted scattering profile of best rigid-body model (solid black line) is overlaid on the measured scattering data ($\chi^2$ = 1.77). Inset: Guinier plot of the low-angle portion of the scattering data are linear consistent with monodisperse solutions. **(b)** Pair-distance distribution function derived from the $\alpha^{UpaB}$ scattering data. **(c)** Comparison between the crystal structure (black cartoon) and the model of the protein structure obtained from *ab initio* modelling against the scattering data. The grey envelope represents the total volume encompassed by the 16 aligned models ($\chi^2$=1.46 ± 0.01 for all models). The probable shape of the protein obtained by the averaging and filtering is shown in magenta. The normalised spatial discrepancy of the 16 models included in the averaging procedure is 0.503 ± 0.013. The ensemble resolution estimated to be 24 ± 2 Å.

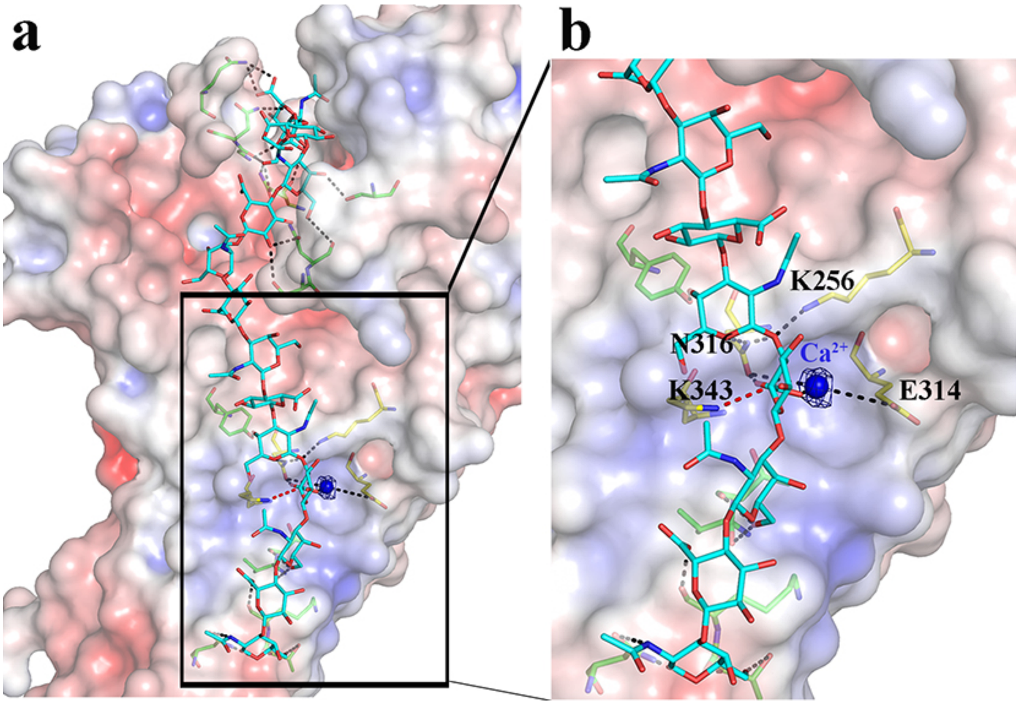


**Supplementary Figure 2:** Molecular docking of a GAG with UpaB. (**a**) Binding mode of a top ranked docked pose of unsulphated chrondroitin into UpaB. For clarity, only interacting residues are displayed in sticks style (green). (**b**) Close-up view of the positioning of the GAG in the groove near the putative the UpaB lyase active site (K256, E314, N316 and K343 are shown in yellow stick representation). Electron density associated with the active site residues modelled as a bound calcium ion is shown (blue sphere/mesh).

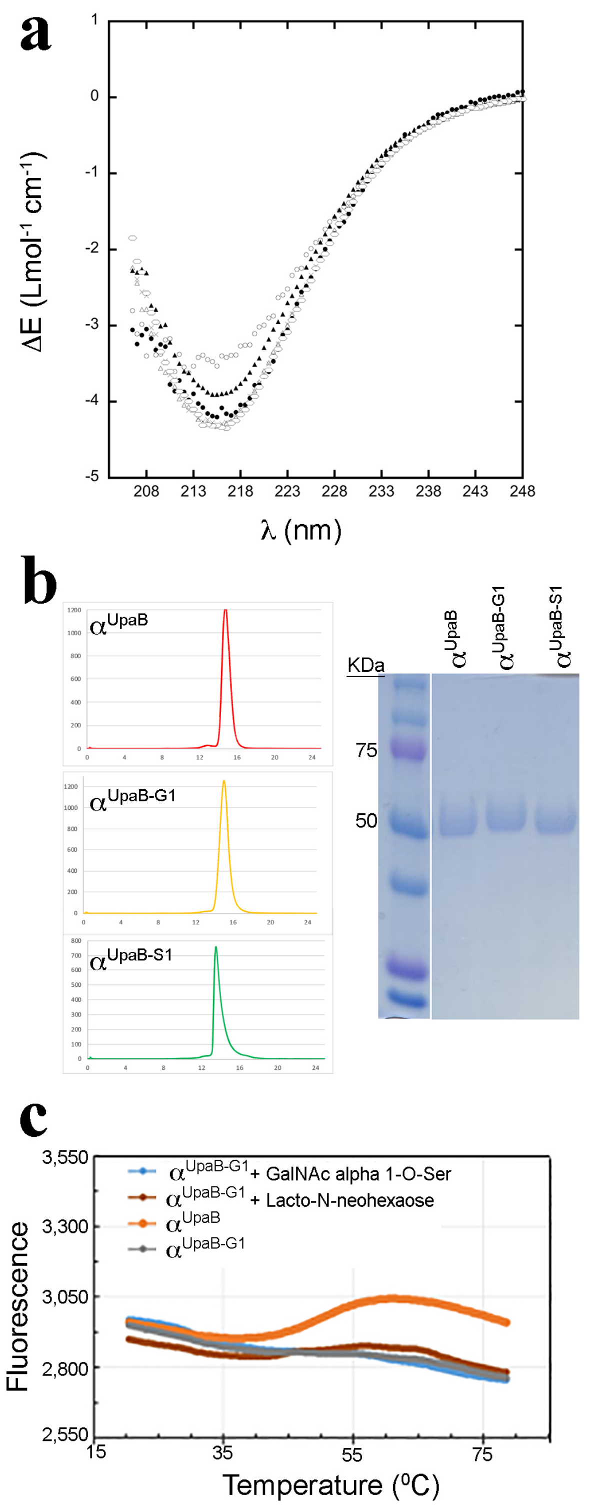


**Supplementary Figure 3:** (**a**) Folding state of UpaB mutants. CD spectral data of $\alpha^{upaB}$(native) (crosses), $\alpha^{UpaB\_G1}$ (empty triangles), $\alpha^{UpaB\text{-}\Delta t1\text{-}2}$ (filled circles), $\alpha^{UpaB\text{-}\Delta t5\text{-}6}$ (empty circles), $\alpha^{UpaB\_S1}$ (filled triangles) and $\alpha^{UpaB\_G1,S1}$ (empty hexagons) mutants at 0.3 mg ml$^{-1}$. Wavelength scans were recorded in the UV region of 200 nm to 250 nm in 0.5 nm increments. (**b**) Elution profile and SDS-PAGE analysis of purified $\alpha^{UpaB}$, $\alpha^{UpaB\_G1}$ and $\alpha^{UpaB\_S1}$. All proteins run through a GE Superdex 10/300 200 pg 24 ml column eluted at 0.6 column volumes with no signs of aggregation. $\alpha^{UpaB}$ native and mutants migrated similarly on SDS-PAGE. (**c**) Melting curve plots showing the fluorescence intensity (relative fluorescence units; RFU) of Sypro orange as a function of temperature for purified $\alpha^{UpaB}$ alone and $\alpha^{UpaB\_G1}$ in the absence and presence of GalN-α1-O-Ser and Lacto-N-neohexaose. The addition of these GAG compounds to $\alpha^{UpaB\_G1}$ did not result in any significant $T_m$ shift relative to the apo protein mutant and native proteins.

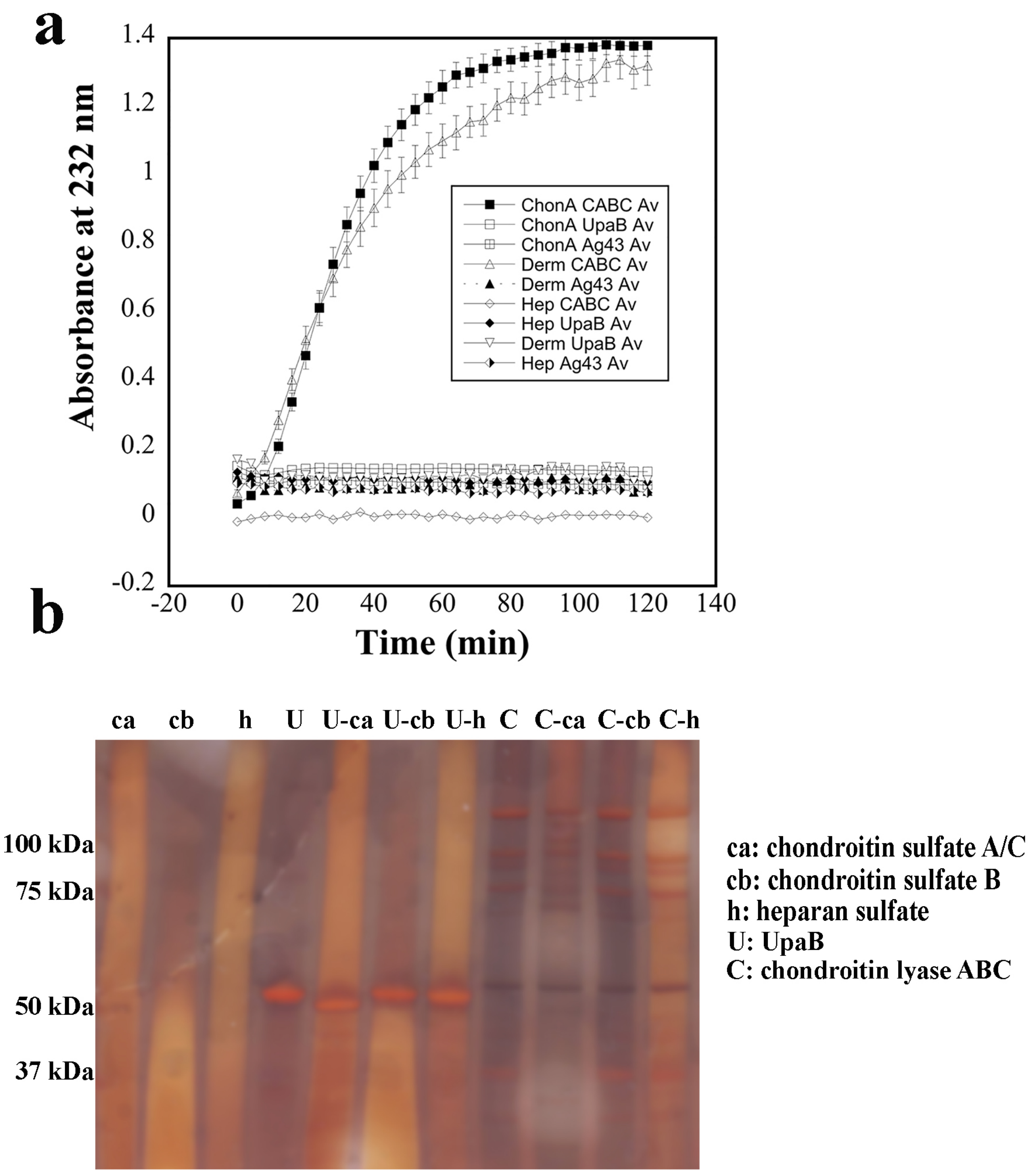


**Supplementary Figure 4: Polysaccharide lyase activity**. (**a**) Polysaccharide lyase assay using UpaB against the glycosaminoglycans Chondroitin sulphate A, B, C and Heparin sulphate. Chondroitin lyase ABC was used as a positive control. The AT Ag43a was used as a negative control. Polysaccharide cleavage causes an increase in $A_{232}$ nm. Data are shown as the means ± standard deviation of 3 replicates. (**b**) Analysis of glycosaminoglycan digests with UpaB. UpaB and Chondroitin lyase ABC were incubated with Chondroitin sulphate A, B, C or Heparin sulphate and then analysed by Gradient (4-12%) SDS-PAGE to detect lyase digestion by Alician Blue/Silver staining. Undigested glycosaminoglycans run as lighter staining smears within the lanes.

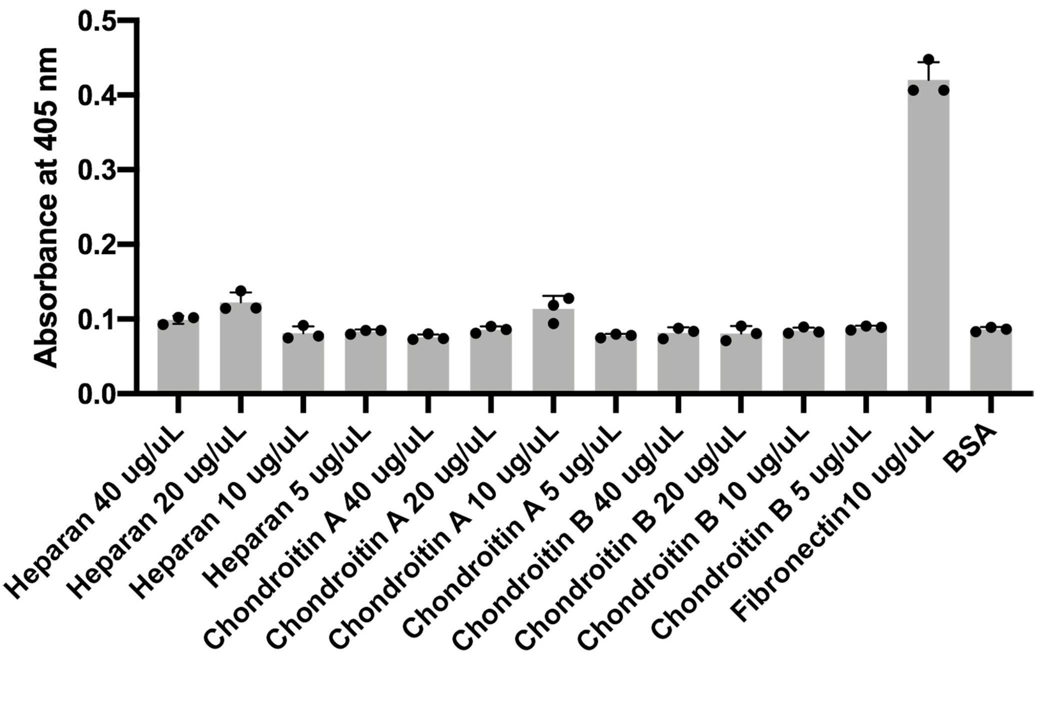


**Supplementary Figure 5: Binding of UpaB to glycosaminoglycans**. Plates were bound with 5-40 μg $ml^{-1}$ of Chondroitin sulfate A, B, C and Heparin sulfate, washed with UpaB and bound UpaB was detected using anti-UpaB polyclonal serum in an ELISA. Binding to fibronectin at 10 μg $ml^{-1}$ was used as a control. The data are expressed as mean ± standard error of the means (SEM) of 3 replicates.

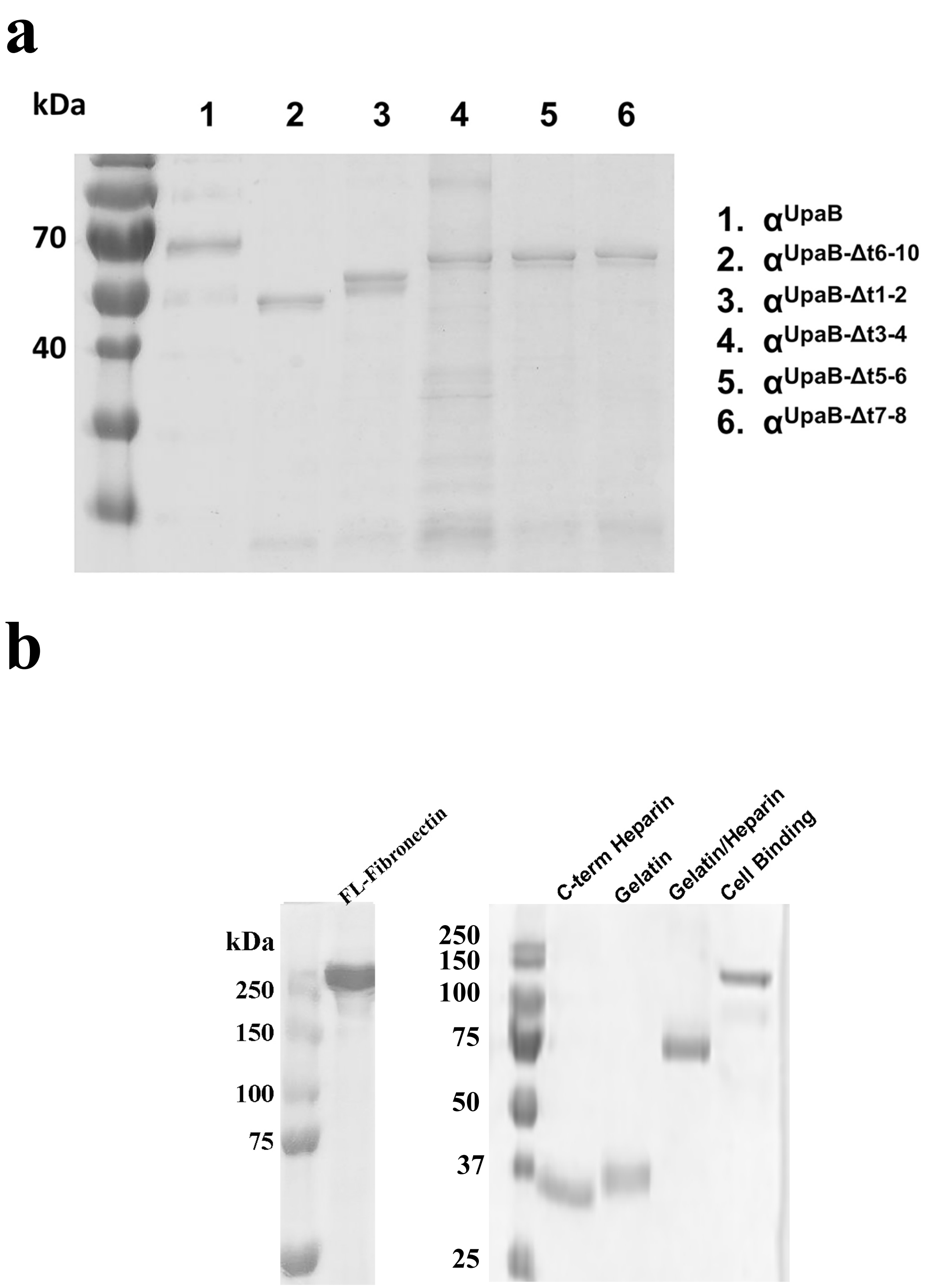


**Supplementary Figure 6:** (**a**) SDS-PAGE analysis of UpaB and UpaB deletion mutant proteins used for ELISAs. Comparable amounts of $\alpha^{UpaB}$(native), $\alpha^{UpaB\text{-}\Delta t6\text{-}10}$, $\alpha^{UpaB\text{-}\Delta t1\text{-}2}$, $\alpha^{UpaB\text{-}\Delta t3\text{-}4}$, $\alpha^{UpaB\text{-}\Delta t5\text{-}6}$ and $\alpha^{UpaB\text{-}\Delta\, t7\text{-}8}$ were used in all assays. (**b**) SDS-PAGE analysis of the purity of full length fibronectin and commercially available fragments used in this work (C-terminal heparin, gelatin, gelatin/heparin, and cell binding domain).

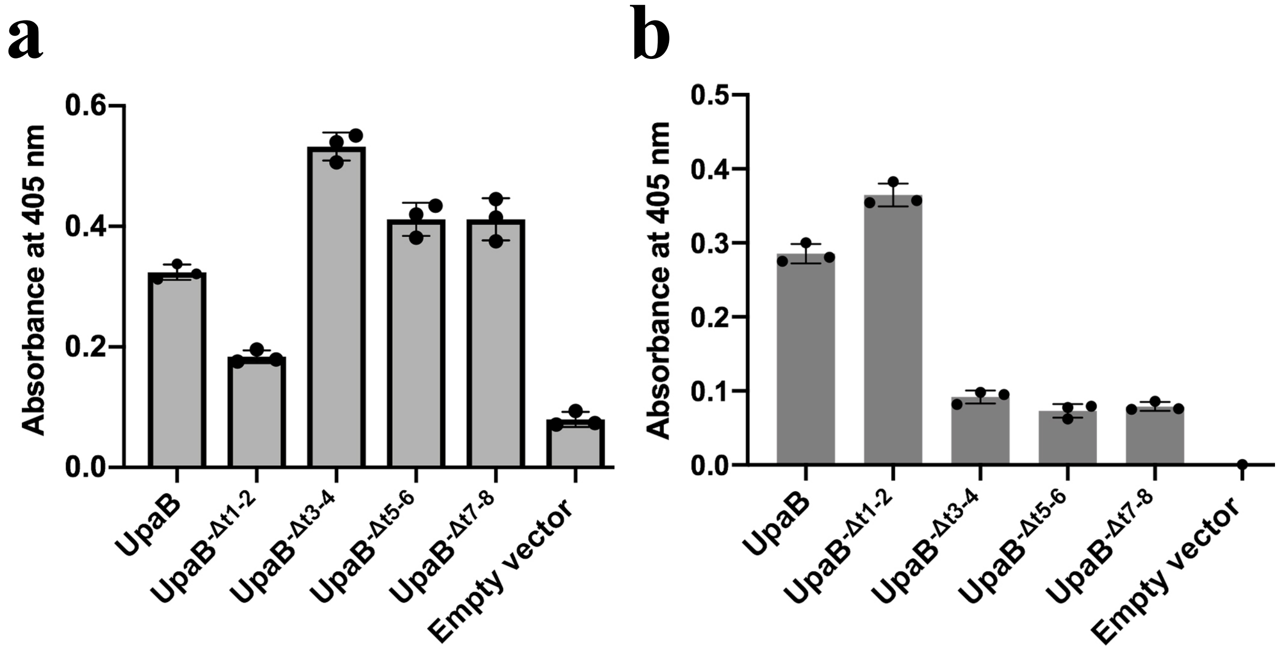


**Supplementary Figure 7:** (**a**) Whole cell ELISA demonstrating expression of full-length UpaB deletion mutants on the *E. coli* cell surface. The following UpaB deletion mutants were examined: UpaB$^{\Delta t1\text{-}2}$, UpaB$^{\Delta t3\text{-}4}$, UpaB$^{\Delta t5\text{-}6}$, UpaB$^{\Delta t7\text{-}8}$ and UpaB (native). Expression of UpaB and UpaB deletion mutants was detected using a UpaB-specific polyclonal antibody. (**b**) Whole cell ELISA demonstrating binding of fibronectin to immobilised *E. coli* cells expressing UpaB or mutant derivatives. The following UpaB deletion mutants were examined: UpaB$^{\Delta t1\text{-}2}$, UpaB$^{\Delta t3\text{-}4}$, UpaB$^{\Delta t5\text{-}6}$, UpaB$^{\Delta t7\text{-}8}$ and UpaB (native). Bound fibronectin was detected using anti-fibronectin antibody in an ELISA. An isogenic control strain containing empty vector pSU2718 was used a negative control. All the data are shown as the means ± standard deviation of 3 replicates.

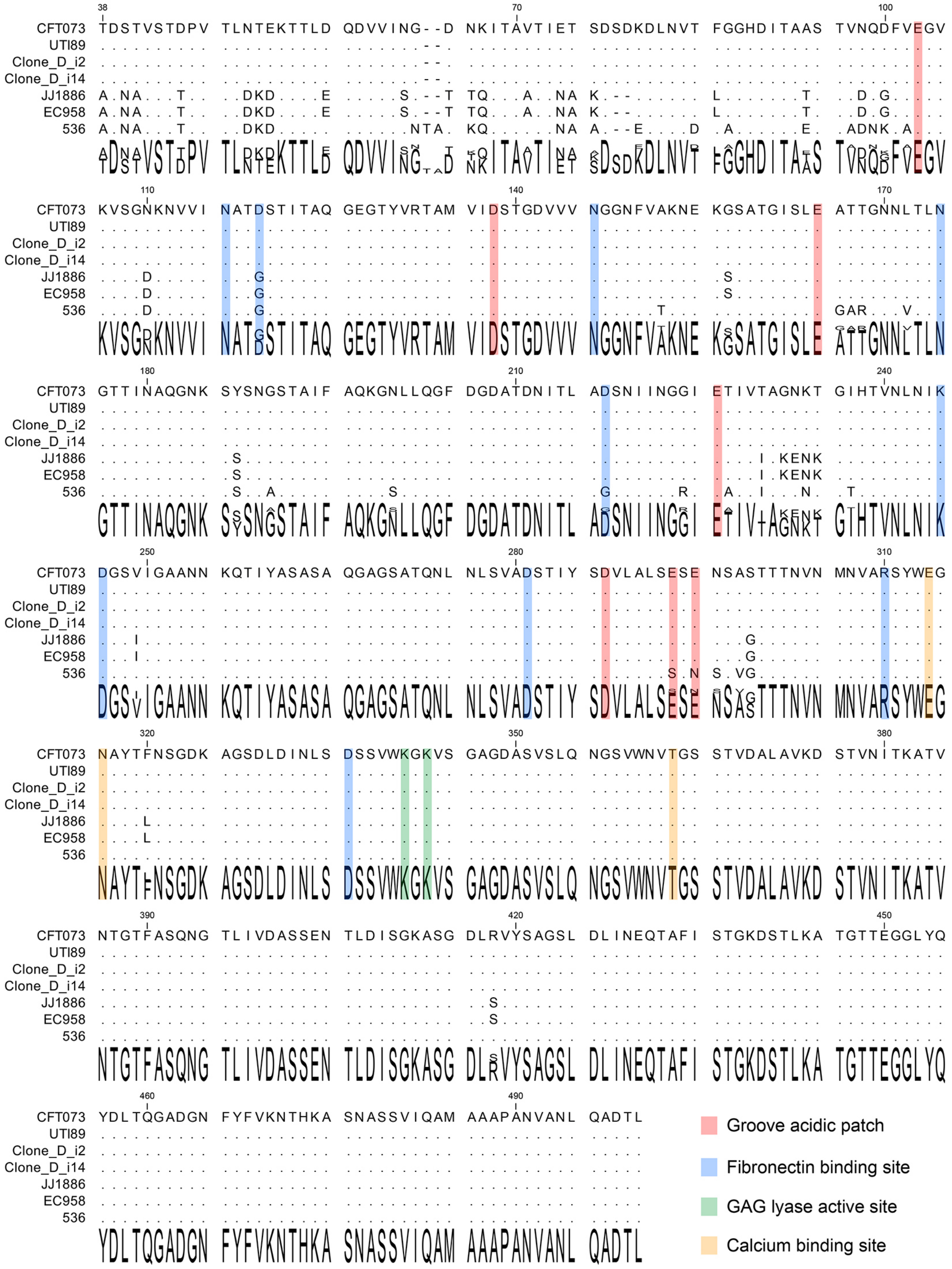

**Supplementary Figure 8:** Comparison of the UpaB clades. Alignment of the five representative UpaB clades with residues that form the FnIII binding site, acidic groove and putative GAG lyase site highlighted.

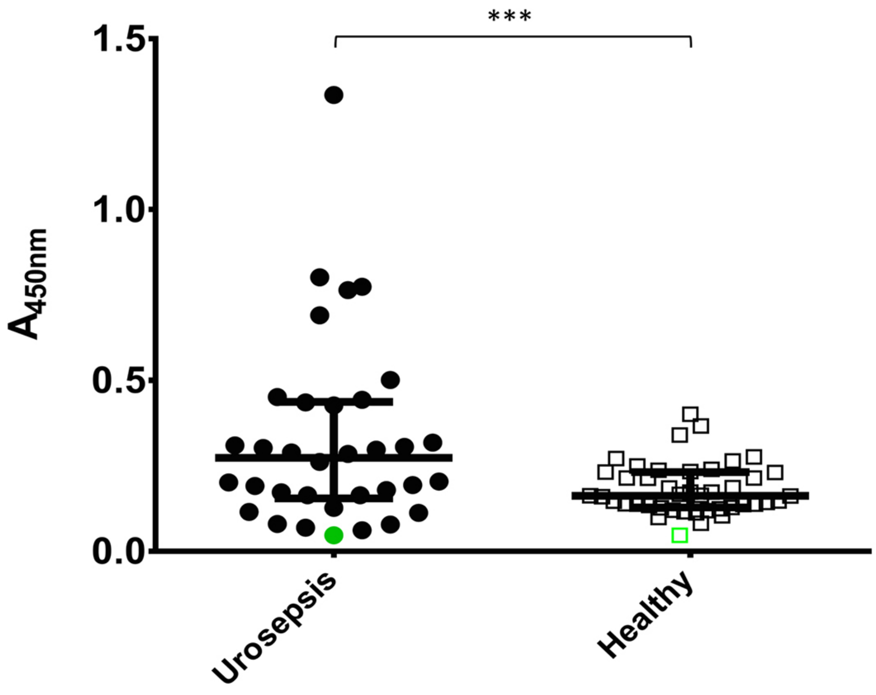


**Supplementary Figure 9:** ELISA to detect anti- $\alpha^{UpaB}$ antibodies from urosepsis patients. Purified $\alpha^{UpaB}$ was used in an ELISA to detect specific anti-UpaG IgG in 33 urosepsis patients compared to 42 healthy individuals. Plasma samples from urosepsis patients infected with UpaB-positive *E. coli* strains produced significantly higher anti-UpaG IgG titers (****P* = 0.0013, unpaired two-sample t-test). Control wells were coated with BSA (filled green circle and green square).

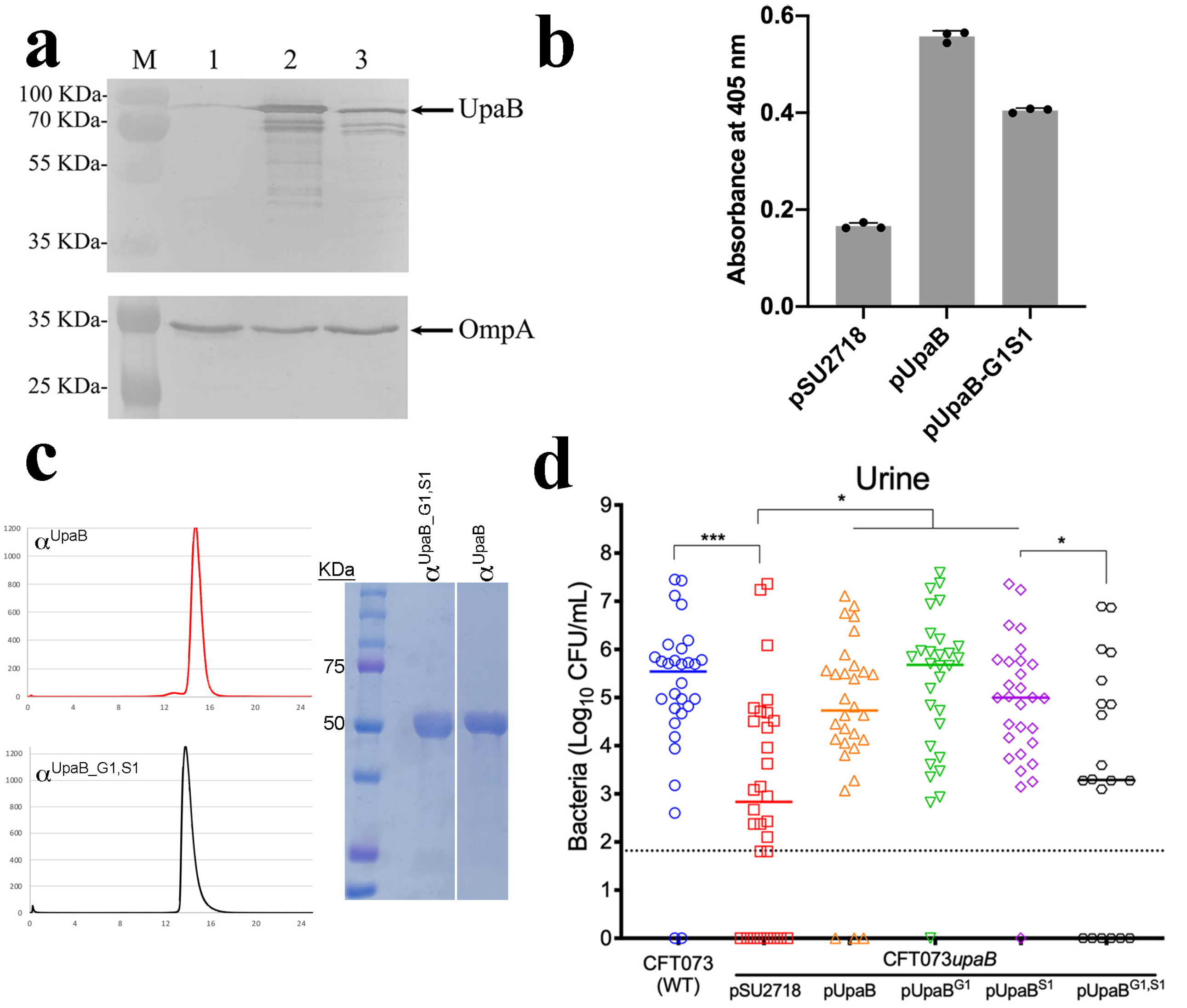


**Supplementary Figure 10:** (**a**) Western blot analysis of whole cell lysates demonstrating the expression of full-length wt UpaB (lane 2) and UpaB$^{G1,S1}$ (lane 3) compared to cells harboring the empty vector (lane 1). The analysis was performed with a UpaB-specific antibody. A band corresponding to the full-length protein was detected, along with minor degradation products. As a loading control to demonstrate equal amounts of protein were examined, a second western blot was performed on the same samples to detect the conserved OmpA protein using an OmpA-specific antibody. Lane M, molecular weight markers. (**b**) Whole cell ELISA confirming the localization of wt UpaB (pUpaB) and UpaB$^{G1,S1}$ (pUpaB-G1,S1) on the bacterial cell surface compared to cells harboring the vector control (pSU2718). Cells from an overnight culture were adsorbed onto a microtitre plate, and specific detection of wt UpaB or UpaB$^{G1,S1}$ was performed using a UpaB-specific antibody. Data are shown as the means ± standard deviation of 3 replicates (**c**) Elution profile and SDS-PAGE analysis of purified $\alpha^{UpaB}$ and $\alpha^{UpaB\_G1,S1}$. Both proteins run through a GE Superdex 10/300 200 pg 24 ml column eluted at 0.6 column volumes with no signs of aggregation. $\alpha^{UpaB}$ native and $\alpha^{UpaB\_G1,S1}$ mutant migrated similarly on SDS-PAGE. (**d**) UPEC persistence in the mouse urinary tract is enhanced by UpaB glycosaminoglycan- and fibronectin-binding interactions. C57BL/6 mice were challenged transurethally with WT CFT073, CFT073*upaB*(pSU2718), CFT073*upaB*(pUpaB), CFT073*upaB*(pUpaB$^{G1}$), CFT073*upaB*(pUpaB$^{S1}$) and CFT073*upaB*(pUpaB$^{G1,\ S1}$). The results represent $\log_{10}$ CFU per ml of urine of individual mice at 24 h post-infection, and the horizontal bars mark group medians. A minimum of 20 mice were assessed per group (pooled from at least 2 independent experiments). Data were compared using Kruskal Wallis analysis of variance (ANOVA) with Dunn's multiple comparisons correction (**P* < 0.05; ****P* < 0.005).

**Supplementary Table 1. SAXS data collection.**

| **Data Collection Parameters** | |
|---|---|
| Instrument | SAXS-WAXS (Australian Synchrotron) |
| Beam geometry | Point |
| Wavelength (Å) | 1.033 |
| $q$-range (Å$^{-1}$) | 0.01-0.60 |
| Exposure time (s) | 35 (35 × 1 sec. exposures) |
| Protein concentration (mg mL$^{-1}$) | 0.7 |
| Temperature (K) | 283 |
| Standard | Water |
| **Structural parameters** | |
| $I(0)$ (cm$^{-1}$) [from Guinier] | 0.02710 ± 0.00006 |
| $R_g$ (Å) [from Guinier] | 29.3 ± 0.1 |
| $I(0)$ (cm$^{-1}$) [from $p(r)$] | 0.02714 ± 0.00005 |
| $R_g$ (Å) [from $p(r)$] | 29.7 ± 0.1 |
| $D_{max}$ (Å) | 105 ± 5 |
| Porod volume (Å$^3$) | 66000 ± 1500 |
| Volume (Å$^3$) [from sequence] | 57300 |
| $R_g$ (Å) [Coral model] | 26.9 |
| $D_{max}$ (Å) [Coral model] | 103 |
| **Molecular mass determination** | |
| Partial specific volume (cm$^3$g$^{-1}$) | 0.725 |
| Contrast, $\Delta\rho$ ($10^{10}$ cm$^{-2}$) | 3.085 |
| Molecular mass $M_r$ [from $I(0)$] | 46700 ± 2500 |
| Molecular mass $M_r$ [from Porod volume] | 54100 ± 2500 |
| Molecular mass $M_r$ [from sequence] | 47700 |
| **Software employed** | |
| Primary data reduction | *Scatterbrain (V2.71)* |
| Data processing | *PRIMUS (V3.2)* [1] *and GNOM (V4.6)* [2] |
| *Ab initio* modelling | *DAMMIN (V5.3)* [3] |
| Validation and averaging | *DAMAVER (V5.0)* [4] |
| Rigid-body modelling | *CORAL (V1.1)* [5] |
| Three-dimensional graphics representations | *PyMOL*[6] |

## Supplementary Table 2. Strains, plasmids and primers used in this study.

| | Relevant description | Source |
|---|---|---|
| **Strains** | | |
| MS427 | MG1655*flu* | [7] |
| BL21(DE3) pLysS | *$F^-$ ompT gal dcm lon hsdSB*(*$rB^-$ $mB^-$*) (*lacIq lacUV5-T7 ind1 sam7 nin5 λcIts857*) | Invitrogen |
| MS5477 | BL21 (DE3) pLysS containing plasmid p$\alpha^{UpaB}$ | This study |
| MS7156 | BL21 (DE3) pLysS containing plasmid p$\alpha^{UpaB_\Delta t6\text{-}10}$ | This study |
| MS7157 | BL21 (DE3) pLysS containing plasmid p$\alpha^{UpaB_\Delta t1\text{-}2}$ | This study |
| MS7158 | BL21 (DE3) pLysS containing plasmid p$\alpha^{UpaB_\Delta t3\text{-}4}$ | This study |
| MS7159 | BL21 (DE3) pLysS containing plasmid p$\alpha^{UpaB_\Delta t5\text{-}6}$ | This study |
| MS7160 | BL21 (DE3) pLysS containing plasmid p$\alpha^{UpaB_\Delta t7\text{-}8}$ | This study |
| MS20219 | BL21 (DE3) pLysS containing plasmid p$\alpha^{UpaB_G1}$ | This study |
| MS8184 | BL21 (DE3) pLysS containing plasmid p$\alpha^{Upa_G2}$ | This study |
| MS8185 | BL21 (DE3) pLysS containing plasmid p$\alpha^{Upa_G3}$ | This study |
| MS8376 | BL21 (DE3) pLysS containing plasmid p$\alpha^{UpaB_S1}$ | This study |
| MS8374 | BL21 (DE3) pLysS containing plasmid p$\alpha^{UpaB_S2}$ | This study |
| MS8375 | BL21 (DE3) pLysS containing plasmid p$\alpha^{UpaB_S3}$ | This study |
| MS4418 | MS427 containing plasmid pSU2718 | This study |
| MS7910 | MS427 containing plasmid pUpaB | This study |
| MS7911 | MS427 containing plasmid pUpaB$_{-}^{\Delta t1\text{-}2}$ | This study |
| MS7912 | MS427 containing plasmid pUpaB$_{-}^{\Delta t3\text{-}4}$ | This study |
| MS7913 | MS427 containing plasmid pUpaB$_{-}^{\Delta t5\text{-}6}$ | This study |
| MS7914 | MS427 containing plasmid pUpaB$_{-}^{\Delta t7\text{-}8}$ | This study |
| MS9736 | MS427 containing plasmid pUpaB$^{G1}$ | This study |
| MS8199 | MS427 containing plasmid pUpaB$^{G2}$ | This study |
| MS8200 | MS427 containing plasmid pUpaB$^{G3}$ | This study |
| MS9737 | MS427 containing plasmid pUpaB$^{S1}$ | This study |
| MS9832 | MS427 containing plasmid pUpaB$^{G1,\ S1}$ | This study |
| MS9717 | CFT073Δ*upaB* containing plasmid pSU2718 | This study |
| MS9718 | CFT073Δ*upaB* containing plasmid pUpaB$^{G1}$ | This study |
| MS9719 | CFT073Δ*upaB* containing plasmid pUpaB$^{S1}$ | This study |
| MS9720 | CFT073Δ*upaB* containing plasmid pUpaB | This study |
| MS9829 | CFT073Δ*upaB* containing plasmid pUpaB$^{G1,\ S1}$ | |
| **Plasmids** | | |
| pLicE | Amp$^r$ | [8] |
| pSU2718 | Cm$^r$ | [9] |
| p$\alpha^{UpaB}$ | *upaB* α-domain in pLicE | This study |
| p$\alpha^{UpaB_\Delta t6\text{-}10}$ | *upaB* α-domain deletion t6-10 (192-343 amino acid residues of αUpaB) in pLicE | This study |
| p$\alpha^{UpaB_\Delta t1\text{-}2}$ | *upaB* α-domain deletion t1-2 (37-97 amino acid residues of αUpaB) in pLicE | This study |
| p$\alpha^{UpaB_\Delta t3\text{-}4}$ | *upaB* α-domain deletion t3-4 (98-156 amino acid residues of αUpaB) in pLicE | This study |
| p$\alpha^{UpaB_\Delta t5\text{-}6}$ | *upaB* α-domain deletion t5-6 (157-222 amino acid residues of αUpaB) in pLicE | This study |
| p$\alpha^{UpaB_\Delta t7\text{-}8}$ | *upaB* α-domain deletion t7-8 (223-285 amino acid residues of αUpaB) in pLicE | This study |
| p$\alpha^{UpaB_G1}$ | *upaB* α-domain (E165A, N189A, Q197A, N200A, Q203A, K256A and N316A) in pLicE | This study |
| p$\alpha^{Upa_G2}$ | *upaB* α-domain (F101A, Y130A, Y187A, F195A, L201G, L202G) in pLicE | This study |
| p$\alpha^{Upa_G3}$ | *upaB* α-domain (E103A, D138A, E165A, E226A) in pLicE | This study |
| p$\alpha^{UpaB_S1}$ | *upaB* α-domain (N116A, D119A, N146A, N175A, D217A, K245A, D246A, D281A, R310A and D336A) in pLicE | This study |
| p$\alpha^{UpaB_S2}$ | *upaB* α-domain (N110A, K111A, N112A, D142A, N171A, D206A, D208A, N212A, N241A, N274A, N276A, N303A, N305A, K325A, D329A, D331A and D349A) in pLicE | This study |
| p$\alpha^{UpaB_S3}$ | *upaB* α-domain (V151A, I221A, V249A, A252G, A253G, Y285A, Y312A and V339A) in pLicE | This study |
| p$\alpha^{UpaB_G1,S1}$ | *upaB* α-domain (E165A, N189A, Q197A, N200A, Q203A, K256A, N316A) and (N116A, D119A, N146A, N175A, D217A, K245A, D246A, D281A, R310A and D336A) in pLicE | This study |
| pUpaB | full length *upaB* in pSU2718 | This study |
| pUpaB$_{-}^{\Delta t1\text{-}2}$ | *upaB* α-domain deletion t1-2 (37-97 amino acid residues of αUpaB) in pSU2718 | This study |
| pUpaB$_{-}^{\Delta t3\text{-}4}$ | *upaB* α-domain deletion t3-4 (98-156 amino acid residues of αUpaB) in pSU2718 | This study |
| pUpaB$_{-}^{\Delta t5\text{-}6}$ | *upaB* α-domain deletion t5-6 (157-222 amino acid residues of αUpaB) in pSU2718 | This study |
| pUpaB$_{-}^{\Delta t7\text{-}8}$ | *upaB* α-domain deletion t7-8 (223-285 amino acid residues of αUpaB) in pSU2718 | This study |

| | | |
|---|---|---|
| pUpaB$^{G1}$ | *upaB* α-domain (E165A, N189A, Q197A, N200A, Q203A, K256A and N316A) in pSU2718 | This study |
| pUpaB$^{G2}$ | *upaB* α-domain (F101A,Y130A,Y187A, F195A, L201G, L202G) in pSU2718 | This study |
| pUpaB$^{G3}$ | *upaB* α-domain (E103A, D138A, E165A, E226A) in pSU2718 | This study |
| pUpaB$^{S1}$ | *upaB* α-domain (N116A, D119A, N146A, N175A, D217A, K245A, D246A, D281A, R310A and D336A) in pSU2718 | This study |
| pUpaB$^{S2}$ | *upaB* α-domain (N110A, K111A, N112A, D142A, N171A, D206A, D208A, N212A, N241A, N274A, N276A, N303A, N305A, K325A, D329A, D331A and D349A) in pSU2718 | This study |
| pUpaB$^{S3}$ | *upaB* α-domain (V151A, I221A, V249A, A252G, A253G, Y285A, Y312A and V339A) in pSU2718 | This study |
| pUpaB$^{G1, S1}$ | *upaB* α-domain (E165A, N189A, Q197A, N200A, Q203A, K256A, N316A) and (N116A, D119A, N146A, N175A, D217A, K245A, D246A, D281A, R310A and D336A) in pSU2718 | This study |

**Supplementary References**


1. Konarev, P.V., Volkov, V.V., Sokolova, A.V., Koch, M.H.J. & Svergun, D.I. PRIMUS: a Windows PC-based system for small-angle scattering data analysis. *Journal of Applied Crystallography* **36**, 1277-1282 (2003).
2. Svergun, D.I. Determination of the regularization parameter in indirect-transform methods using perceptual criteria. *J. Appl. Cryst.* **25**, 495-503 (1992).
3. Svergun, D.I. Restoring low resolution structure of biological macromolecules from solution scattering using simulated annealing. *Biophys J* **76**, 2879-86 (1999).
4. Volkov, V.V. & Svergun, D.I. Uniqueness of ab initio shape determination in small-angle scattering. *Journal of Applied Crystallography* **36**, 860-864 (2003).
5. Petoukhov, M.V. et al. New developments in the ATSAS program package for small-angle scattering data analysis. *J Appl Crystallogr* **45**, 342-350 (2012).
6. DeLano, W.L. The PyMOL Molecular Graphics System, http://www.pymol.org . *DeLano Scientific, San Carlos, CA, USA.* (2002).
7. Reisner, A., Haagensen, J.A., Schembri, M.A., Zechner, E.L. & Molin, S. Development and maturation of Escherichia coli K-12 biofilms. *Mol Microbiol* **48**, 933-46 (2003).
8. Eschenfeldt, W.H., Lucy, S., Millard, C.S., Joachimiak, A. & Mark, I.D. A family of LIC vectors for high-throughput cloning and purification of proteins. *Methods Mol Biol* **498**, 105-15 (2009).
9. Martinez, E., Bartolome, B. & de la Cruz, F. pACYC184-derived cloning vectors containing the multiple cloning site and lacZ alpha reporter gene of pUC8/9 and pUC18/19 plasmids. *Gene* **68**, 159-62 (1988).
