# Supplementary material for: Unique structural features of a bacterial autotransporter adhesin suggest mechanisms for interaction with host macromolecules
Source: Nat Commun. 2019 Apr 29;10:1967. doi: 10.1038/s41467-019-09814-6 (PMC6488583; doi:10.1038/s41467-019-09814-6)
Supplement: Supplementary file 2 — Description of Additional Supplementary Files [file 41467_2019_9814_MOESM2_ESM.pdf]

Description of Additional Supplementary Files

File Name: Supplementary Movie 1

Description: Proposed model for the interaction between UpaB and FnIII. Modelling simulations were performed between the crystal structures of α^{UpaB} and $FnIII_{1-2}$ (2HA1) using NAMD with 3 x 400 nanosecond simulations (1.2 microseconds total). α^{UpaB} residues experimentally identified as part of the Fn binding interface (N116, D119, N146, N175, D217, K245, D246, D281, R310 and D336) are shown in stick representation and highlighted with transparent red spheres. Interacting residues belonging to the α^{UpaB} – $FnIII_{1-2}$ interface during the simulations are highlighted by stick representations and transparent blue spheres. Predominant hydrogen bond interactions are observed between the binding interface and charged residues on $FnIII_{1-2}$.

File Name: Supplementary Movie 2

Description: Modelling of the interaction between α^{UpaB} S1 mutant and FnIII. Modelling simulations were performed between the modified crystal structure of α^{UpaB} incorporating N116A, D119A, N146A, N175A, D217A, K245A, D246A, D281A, R310A and D336A mutations and $FnIII_{1-2}$ (2HA1) using NAMD for 3 x 400 nanosecond simulations (1.2 microseconds total). Mutated residues are highlighted with transparent red spheres. Interacting residues belonging to the α^{UpaB} S1 – $FnIII_{1-2}$ interface during the simulations are highlighted by stick representations and blue spheres. Abolishing the charged residues of the binding interface of UpaB greatly reduces the interaction with $FnIII_{1-2}$.
