# Supplementary material for: Unique structural features of a bacterial autotransporter adhesin suggest mechanisms for interaction with host macromolecules
Source: Nat Commun. 2019 Apr 29;10:1967. doi: 10.1038/s41467-019-09814-6 (PMC6488583; doi:10.1038/s41467-019-09814-6)
Supplement: Supplementary file 5 — Reporting Summary [file 41467_2019_9814_MOESM5_ESM.pdf]

nature research

Corresponding author(s): Begona Heras
Mark Schembri

Last updated by author(s): Mar 19, 2019

Reporting Summary

Nature Research wishes to improve the reproducibility of the work that we publish. This form provides structure for consistency and transparency in reporting. For further information on Nature Research policies, see Authors & Referees and the Editorial Policy Checklist.

Statistics

For all statistical analyses, confirm that the following items are present in the figure legend, table legend, main text, or Methods section.

n/a	Confirmed	
☐	☒	The exact sample size (*n*) for each experimental group/condition, given as a discrete number and unit of measurement
☐	☒	A statement on whether measurements were taken from distinct samples or whether the same sample was measured repeatedly
☐	☒	The statistical test(s) used AND whether they are one- or two-sided<br>*Only common tests should be described solely by name; describe more complex techniques in the Methods section.*
☐	☒	A description of all covariates tested
☒	☐	A description of any assumptions or corrections, such as tests of normality and adjustment for multiple comparisons
☐	☒	A full description of the statistical parameters including central tendency (e.g. means) or other basic estimates (e.g. regression coefficient) AND variation (e.g. standard deviation) or associated estimates of uncertainty (e.g. confidence intervals)
☐	☒	For null hypothesis testing, the test statistic (e.g. *F*, *t*, *r*) with confidence intervals, effect sizes, degrees of freedom and *P* value noted<br>*Give P values as exact values whenever suitable.*
☒	☐	For Bayesian analysis, information on the choice of priors and Markov chain Monte Carlo settings
☒	☐	For hierarchical and complex designs, identification of the appropriate level for tests and full reporting of outcomes
☒	☐	Estimates of effect sizes (e.g. Cohen's *d*, Pearson's *r*), indicating how they were calculated

Our web collection on statistics for biologists contains articles on many of the points above.

Software and code

Policy information about availability of computer code

Data collection	Diffraction data were collected using the Blue Ice software. (McPhillips et al. J. Synchrotron Rad. (2002). 9, 401-406)
Data analysis	All software used was commercial and used by standard specifications<br><br>Protein structural alignments<br>- DALI server (http://ekhidna.biocenter.helsinki.fi/dali_server/start)<br><br>Protein structure determination by X-ray crystallography<br>- Data were integrated, scaled and merged using HKL2000 (Otwinowski, Z. & Minor, W. Processing of X-ray diffraction data collected in oscillation mode. Methods Enzymol 276, 307-326 1997) and XDS/XSCALE (Kabsch, W. Xds. Acta Crystallogr D Biol Crystallogr 66, 125-32 2010).<br>- Structure was solved using SHELX C,D,E (Sheldrick, G.M. Experimental phasing with SHELXC/D/E: combining chain tracing with density modification. Acta Crystallogr D Biol Crystallogr 66, 479-85 2010).<br>- Model building ARP/wARP (Langer, G., Cohen, S.X., Lamzin, V.S. & Perrakis, A. Automated macromolecular model building for X-ray crystallography using ARP/wARP version 7. Nat Protoc 3, 1171-9 2008) and COOT (Emsley, P. & Cowtan, K. Coot: model-building tools for molecular graphics. Acta Crystallogr D Biol Crystallogr 60, 2126-32 2004).<br>- Protein structure refinement Refmac 5 (Murshudov, G.N. et al. REFMAC5 for the refinement of macromolecular crystal structures. Acta Crystallogr D Biol Crystallogr 67, 355-67 2011).<br>and Phenix.refine (Adams, P.D. et al. PHENIX: building new software for automated crystallographic structure determination. Acta Crystallogr D Biol Crystallogr 58, 1948-54 2002).<br>- Protein structure validation MolProbity (Davis, I.W. et al. MolProbity: all-atom contacts and structure validation for proteins and nucleic acids. Nucleic Acids Res 35, W375-83 2007).<br>- Protein structure figures were created with PyMOL (DeLano, W.L. The PyMOL Molecular Graphics System, http://www.pymol.org/ . DeLano Scientific, San Carlos, CA, USA. 2002).

Analytical Ultracentrifugation
- Solvent density, solvent viscosity and partial specific volume estimates calculated using SEDNTERP (Lebowitz, J., Lewis, M.S. & Schuck, P. Modern analytical ultracentrifugation in protein science: a tutorial review. Protein Sci 11, 2067-79 2002).
- Data were fitted SEDFIT (Schuck, P. et al. SEDFIT-MSTAR: molecular weight and molecular weight distribution analysis of polymers by sedimentation equilibrium in the ultracentrifuge. Analyst 139, 79-92 2014).

SAXS Analysis
- Data correction and averaging used Scatterbrain v 2.7.1 (www.synchrotron.org.au)
- Molecular masses estimated using MULCh v 1.1 (Whitten, A.E., Cai, S. & Trewhella, J. MULCh: modules for the analysis of small-angle neutron contrast variation data from biomolecular assemblies. J. Appl. Cryst. 41, 222-226 2008).
- Data processing and Guinier analysis performed using Primus v 3.2 (Konarev, P.V., Volkov, V.V., Sokolova, A.V., Koch, M.H.J. & Svergun, D.I. PRIMUS: a Windows PC-based system for small-angle scattering data analysis. Journal of Applied Crystallography 36, 1277-1282 2003).
- Pair-distance distribution function calculated using GNOM v 4.6 (Svergun, D.I. Determination of the regularization parameter in indirect-transform methods using perceptual criteria. J. Appl. Cryst. 25, 495-503 1992).
- Models of the data were created using CORAL v 1.1 (Petoukhov, M.V. et al. New developments in the ATSAS program package for small-angle scattering data analysis. J Appl Crystallogr 45, 342-350 2012).
- Molecular envelopes were created using DAMMIN v 5.3 (Svergun, D.I. Restoring low resolution structure of biological macromolecules from solution scattering using simulated annealing. Biophys J 76, 2879-86 1999).
- Molecular envelopes were filtered using DAMAVER v 2.8 (Volkov, V.V. & Svergun, D.I. Uniqueness of ab initio shape determination in small-angle scattering. Journal of Applied Crystallography 36, 860-864 2003).
- Deposition of SAXS data in SASBDB (Valentini, E., Kikhney, A.G., Previtali, G., Jeffries, C.M. & Svergun, D.I. SASBDB, a repository for biological small-angle scattering data. Nucleic Acids Res 43, D357-63 2015).
Fluorescence thermal shift assays
Flurorescence data fitting Excel XLfit (www.microsoft.com)

Sequence Analysis
CLC Main Workbench Qiagen (https://www.qiagenbioinformatics.com/).

Modelling
- dynamics simulations used NAMD2.12 (Phillips, J.C. et al. Scalable molecular dynamics with NAMD. J Comput Chem 26, 1781-802 2005).
- viewing and mapping simulations used VMD (Humphrey, W., Dalke, A. & Schulten, K. VMD: visual molecular dynamics. J Mol Graph 14, 33-8, 27-8 1996).
- docking used Autodock Vina (Trott, O. & Olson, A.J. AutoDock Vina: improving the speed and accuracy of docking with a new scoring function, efficient optimization, and multithreading. J Comput Chem 31, 455-61 2010).

For manuscripts utilizing custom algorithms or software that are central to the research but not yet described in published literature, software must be made available to editors/reviewers. We strongly encourage code deposition in a community repository (e.g. GitHub). See the Nature Research guidelines for submitting code & software for further information.

Data

Policy information about availability of data

All manuscripts must include a data availability statement. This statement should provide the following information, where applicable:
- Accession codes, unique identifiers, or web links for publicly available datasets
- A list of figures that have associated raw data
- A description of any restrictions on data availability

The data that support the findings of this study are available from the corresponding authors upon request. Coordinates and structure-factor files have been deposited in the Protein Data Bank, with accession code 6BEA. Scattering data and models have been deposited in the SASBDB with accession code SASDC45.

Field-specific reporting

Please select the one below that is the best fit for your research. If you are not sure, read the appropriate sections before making your selection.

☒ Life sciences ☐ Behavioural & social sciences ☐ Ecological, evolutionary & environmental sciences

For a reference copy of the document with all sections, see nature.com/documents/nr-reporting-summary-flat.pdf

Life sciences study design

All studies must disclose on these points even when the disclosure is negative.

Sample size	An arbitrary sample size of 45 patients was selected for urine study<br><br>A minimum of 20 mice were assessed per group

Data exclusions	No data was excluded from this manuscript
Replication	All ELISA experimental data are the means ± standard deviation of 3 replicates.<br>AUC and CD experiments were performed in triplicate. Only one representative graph is shown in the manuscript.<br>Molecular dynamics is a cumulative result of three independent simulations.<br>Docking involved 9 independent simulations and the top hit was selected based upon binding energy.
Randomization	In ELISA to detect anti- αUpaB antibodies from urosepsis patients two experimental groups were defined, based upon professional medical diagnosis of urosepsis at the Princess Alexandra Hospital (Brisbane, Australia).<br>Mouse data was obtained from at least 20 mice per group, pooled from at least 2 independent experiments.
Blinding	N/A

Reporting for specific materials, systems and methods

We require information from authors about some types of materials, experimental systems and methods used in many studies. Here, indicate whether each material, system or method listed is relevant to your study. If you are not sure if a list item applies to your research, read the appropriate section before selecting a response.

Materials & experimental systems

n/a	Involved in the study	
☐	☒	Antibodies
☒	☐	Eukaryotic cell lines
☒	☐	Palaeontology
☐	☒	Animals and other organisms
☐	☒	Human research participants
☒	☐	Clinical data

Methods

n/a	Involved in the study	
☒	☐	ChIP-seq
☒	☐	Flow cytometry
☒	☐	MRI-based neuroimaging

Antibodies

Antibodies used	Rabbit polyclonal serum against UpaB (WEHI antibody facility, Melbourne, Australia),<br>Alkaline phosphatase-conjugated goat anti-rabbit IgG (SIGMA, A3687, Lot#SLBK3154V)<br>Anti-fibronectin antibody (Sigma, F3648, , Lot#103M4818V)<br>Peroxidase-conjugated anti-human IgG (SIGMA, A8667)
Validation	Rabbit polyclonal serum against UpaB (WEHI antibody facility, Melbourne, Australia), validated by Wetern Blotting using purified recombinant functional alpha-domain of UpaB.<br><br>Alkaline phosphatase-conjugated goat anti-rabbit IgG (SIGMA, A3687, Lot#SLBK3154V)<br>Commercially tested applications: immunoblotting 1:30,000, immunohistochemistry (formalin-fixed, paraffin-embedded sections) 1:50,direct ELISA 1:30,000, Anti Rabbit Sigma, anti rabbit antibody<br><br>Anti-fibronectin antibody (Sigma, F3648, , Lot#103M4818V) Commercially tested applications: microarray suitable, immunoblotting human plasma fibronectin 1:1,000, indirect immunofluorescence human foreskin cultured fibroblasts 1:400,indirect ELISA, Fibronectin Antibody Sigma, Fibronectin Antibody<br><br>Peroxidase-conjugated anti-human IgG (SIGMA, A8667)<br>Commercially tested applications: Goat Anti-Human IgG (whole molecule)-Peroxidase antibody has been used for ELISA]. The product can also be used for dot blot (1:80,000) and immunohistochemistry (1:200).

Animals and other organisms

Policy information about studies involving animals; ARRIVE guidelines recommended for reporting animal research

Laboratory animals	C57BL/6 female mice aged 10-12 weeks. Source: Animal Resources Centre, Canning Vale, Western Australia 6970, Australia.
Wild animals	n/a
Field-collected samples	n/a
Ethics oversight	All animal experimentation was conducted in accordance with the guidelines of the National Health and Medical Research Council. The Griffith University Animal Ethics Committee approved this study (MSC/01/18/AEC).

Note that full information on the approval of the study protocol must also be provided in the manuscript.

Human research participants

Policy information about studies involving human research participants

Population characteristics	Patients with urosepsis were included in the study.
Recruitment	Patients with urosepsis were included in the study if the same Gram-negative organism was cultured from their blood and urine at the time of admission to the Princess Alexandra Hospital (Brisbane, Australia).
Ethics oversight	The use of human blood plasma from patients was approved by the institutional review board of the Princess Alexandra Hospital (2008/264). The need for patient informed consent was waived, as the primary purpose for the collection of these samples was for other diagnostic procedures, and all patient information was de-identified. The collection of human blood from control subjects was approved by the institutional review board of Griffith University (MSC/18/10/HREC). Informed consent was obtained from all control subjects.

Note that full information on the approval of the study protocol must also be provided in the manuscript.
